# Managing Friends and Foes: Sanctioning Mutualists in Mixed‐Infection Nodules Trades off With Defense Against Antagonists

**DOI:** 10.1111/eva.70064

**Published:** 2024-12-29

**Authors:** Camille E. Wendlandt, Saumik Basu, Angeliqua P. Montoya, Paige Roberts, Justin D. Stewart, Allison B. Coffin, David W. Crowder, E. Toby Kiers, Stephanie S. Porter

**Affiliations:** ^1^ School of Biological Sciences Washington State University Vancouver Washington USA; ^2^ Department of Entomology Washington State University Pullman Washington USA; ^3^ Department of Entomology University of Georgia Tifton Georgia USA; ^4^ Amsterdam Institute for Life and Environment (A‐LIFE), Section Ecology & Evolution Vrije Universiteit Amsterdam Amsterdam The Netherlands; ^5^ Department of Integrative Physiology and Neuroscience Washington State University Vancouver Washington USA

**Keywords:** aphids, legume‐rhizobia mutualism, mutualism management, Pea Enation Mosaic Virus, sanctions, symbiosis

## Abstract

Successful plant growth requires plants to minimize harm from antagonists and maximize benefit from mutualists. However, these outcomes may be difficult to achieve simultaneously, since plant defenses activated in response to antagonists can compromise mutualism function, and plant resources allocated to defense may trade off with resources allocated to managing mutualists. Here, we investigate how antagonist attack affects plant ability to manage mutualists with sanctions, in which a plant rewards cooperative mutualists and/or punishes uncooperative mutualists. We studied interactions among wild and domesticated pea plants, pea aphids, an aphid‐vectored virus (Pea Enation Mosaic Virus, PEMV), and mutualistic rhizobial bacteria that fix nitrogen in root nodules. Using isogenic rhizobial strains that differ in their ability to fix nitrogen and express contrasting fluorescent proteins, we found that peas demonstrated sanctions in both singly‐infected nodules and mixed‐infection nodules containing both strains. However, the plant's ability to manage mutualists in mixed‐infection nodules traded off with its ability to defend against antagonists: when plants were attacked by aphids, they stopped sanctioning within mixed‐infection nodules, and plants that exerted stricter sanctions within nodules during aphid attack accumulated higher levels of the aphid‐vectored virus, PEMV. Our findings suggest that plants engaged in defense against antagonists suffer a reduced ability to select for the most beneficial symbionts in mixed‐infection tissues. Mixed‐infection tissues may be relatively common in this mutualism, and reduced plant sanctions in these tissues could provide a refuge for uncooperative mutualists and compromise the benefit that plants obtain from mutualistic symbionts during antagonist attack. Understanding the conflicting selective pressures plants face in complex biotic environments will be crucial for breeding crop varieties that can maximize benefits from mutualists even when they encounter antagonists.

## 1 Introduction

In both natural and agricultural environments, plant performance is shaped by the benefits gained from microbial mutualists, such as rhizobia and mycorrhizal fungi, and the harm imposed by antagonists, such as herbivores and pathogens. When interacting with mutualists, plants can manage their interactions to preferentially exchange resources with superior partners. For instance, plants can use sanctions to reward more beneficial symbionts with carbon based on their level of cooperation (Bever et al. 2009; Jander and Herre 2016; Westhoek et al. 2021) and to punish less beneficial symbionts by selectively inducing senescence of the plant cells they inhabit (Regus et al. 2017). When attacked by antagonists, plants can deploy defenses such as localized plant cell death, increased production of physical and chemical defenses, and release of volatiles that recruit enemies of their antagonists (Basu et al. 2021; Mostafa et al. 2022). However, little research addresses how well plants use sanctions on mutualists when they are simultaneously responding to attacks by antagonists (Siepielski and Benkman 2010). If sanctions change in response to antagonist attacks, this could impact how well plants maximize benefits from mutualists in natural and agricultural environments, where antagonists are common.

In the legume‐rhizobia mutualism, legumes recruit mutualistic rhizobium soil bacteria to infect root tissue, differentiating these infected regions into root nodules (Poole, Ramachandran, and Terpolilli 2018) where rhizobia fix atmospheric nitrogen into reduced forms that the plant can use (Ledermann, Schulte, and Poole 2021). Nitrogen fixation by rhizobia can meet a substantial portion of the plant's nitrogen needs, making legumes some of the most agronomically important plants in the world (Stagnari et al. 2017) and one of the primary biological sources of nitrogen in terrestrial environments (Reed, Cleveland, and Townsend 2011; Vitousek et al. 2013). Legumes can manage infection by rhizobial mutualists via sanctions, in which legumes preferentially confer resources to rhizobia that are conferring greater net benefits, causing the nodules and plant cells housing these superior mutualists to be larger and better provisioned than those housing less beneficial strains (Kiers et al. 2003; Oono, Anderson, and Denison 2011; Regus et al. 2017; Westhoek et al. 2017, 2021; Montoya et al. 2023). Sanctions also include punitive measures against less beneficial mutualists, including divestment of resources (Kiers et al. 2003) and active senescence of plant cells bearing less‐beneficial symbionts (Regus et al. 2017; Westhoek et al. 2021). Here, we use the term “sanctions” broadly to refer to plants treating symbionts differently based on their level of cooperation, regardless of whether this involves rewarding more‐beneficial symbionts or punishing less‐beneficial symbionts. Sanctions are considered an adaptive trait that evolves in response to abundant natural variation in symbiotic partner quality, allowing nodule‐forming legumes to optimize their benefits from diverse populations of rhizobia (Porter et al. 2024).

**FIGURE 1:**
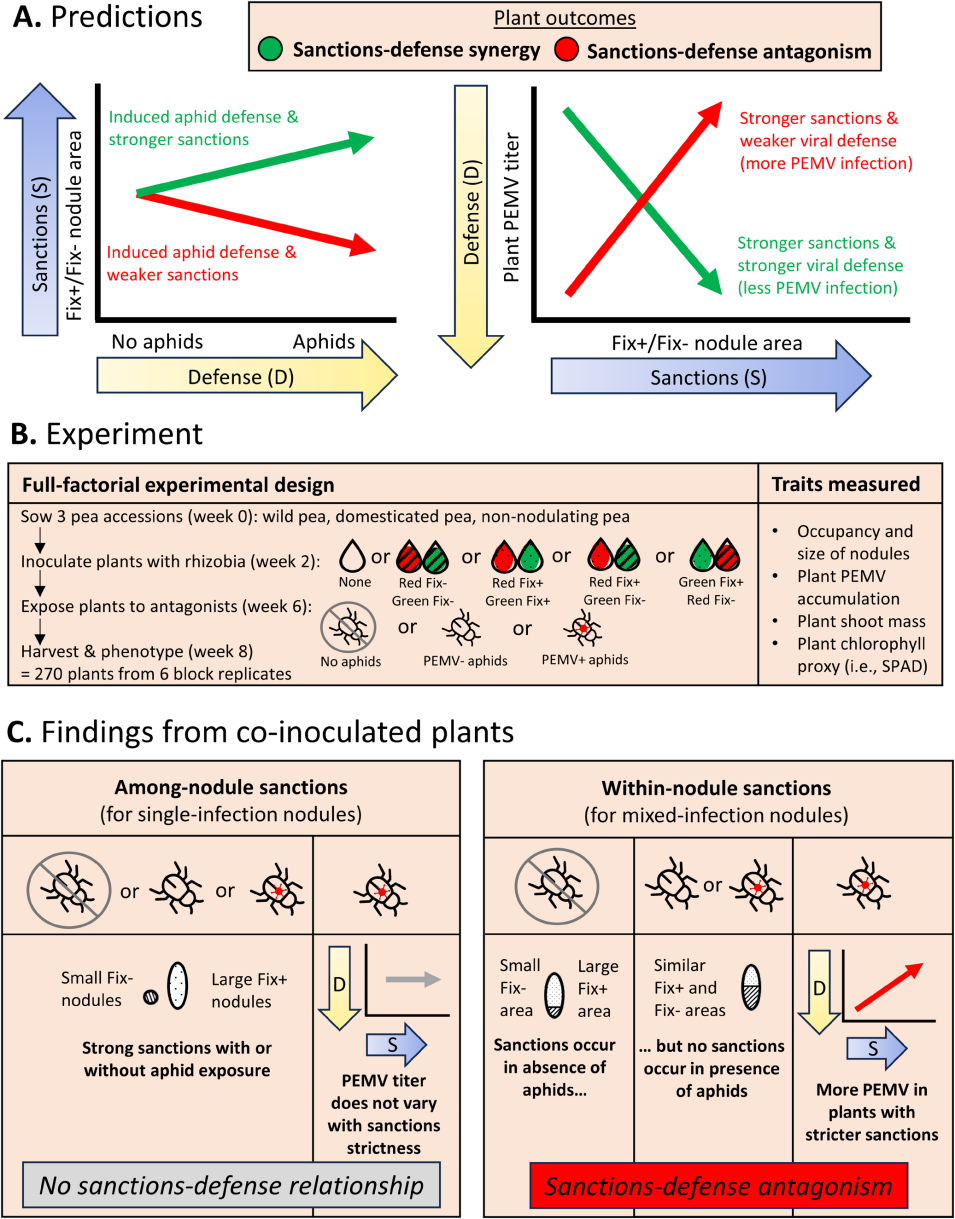
Predictions, experimental design, and main findings. (A) Predictions of how sanctions against Fix− rhizobia interact with plant defense under two hypotheses (sanctions‐defense synergy and sanctions‐defense antagonism). We measured sanctions by comparing the root nodule area allocated to Fix+ versus Fix− rhizobia, with greater relative investment in Fix+ rhizobia corresponding to stricter sanctions. We measured sanctions separately for a plant's singly‐infected nodules (i.e., among‐nodule sanctions) and mixed‐infection nodules (i.e., within‐nodule sanctions). (B) Experimental design highlighting fully‐crossed experimental treatments and timeline of each treatment. (C) Main experimental findings at harvest: pea plants showed among‐nodule sanctions regardless of aphid treatment, and PEMV accumulation did not vary with among‐nodule sanctions, consistent with no sanctions‐defense relationship. However, within‐nodule sanctions disappeared when plants were exposed to aphids, and individual plants with stricter sanctions accumulated more PEMV, consistent with sanctions‐defense antagonism.

Despite the performance benefits legumes can obtain from rhizobial symbionts, legume productivity is commonly constrained by antagonists like sap‐feeding aphids and aphid‐vectored viruses (Jones 2012; Basu et al. 2021; Mofokeng and Gerrano 2021). Antagonists can have complex effects on legume symbiosis with rhizobia: plants can increase nodule formation after exposure to natural herbivores (Cassidy et al. 2022) or reduce nodule formation after exposure to aphids (Basu et al. 2021) and root‐knot nematodes (Wood et al. 2018; Burr et al. 2022). However, a tradeoff between defense and sanctions seems likely, given how these mechanisms both incur physiological costs for plants (West, Kiers, and Pen 2002; Tian et al. 2003), such as the targeted degradation of plant tissue containing antagonists or uncooperative symbionts, respectively (Dickman and Fluhr 2013; Regus et al. 2017; Serova, Tsyganova, and Tsyganov 2018). If antagonist attack shifts resources away from sanctions, then plants could show weaker sanctions when they are defending themselves from antagonists compared to when they are free from antagonists (Figure 1A). Other work on legumes has showed how the benefits from mutualists can alter when plants are exposed to antagonists (Simonsen and Stinchcombe 2014a; Wood et al. 2018), but our work is novel in that we link the effects of antagonists to plant sanctioning of mutualists.

Plant defense against antagonists involves several interacting signaling pathways (Kunkel and Brooks 2002). Even closely related microbial strains can trigger different plant immune responses, leading to dramatically different consequences for a plant's resistance to different types of antagonists (Haney et al. 2017). When multiple microbial strains are present on a plant, there can be strong regulatory cross‐talk among different activated defense responses—jasmonic acid (JA)‐ and ethylene (ET)‐dependent pathways tend to function synergistically, enhancing each other's function, and salicylic acid (SA)‐ and JA‐dependent pathways function antagonistically, attenuating each other's function (Kunkel and Brooks 2002)—which may lead to unexpected outcomes for the plant‐antagonist interaction (Tonelli, Magallanes‐Noguera, and Fabra 2017). These plant defense pathways are also important players at the onset of symbiosis, when microbial mutualists must sequentially downregulate components of the host immune system to establish a symbiotic population (Cao et al. 2017; Porter et al. 2024). Furthermore, after symbiosis is established, plant phytohormones continue to impact both defense from nematodes and maintenance of the rhizobial symbiosis (Costa, Ng, and Mathesius 2021), and legumes with SA‐dependent defense induced by aphids show reduced function of their nitrogen‐fixing rhizobial symbionts (Basu et al. 2021).

Genes with dual roles in defense and symbiosis provide additional insights into mechanisms by which defense from antagonists can constrain mutualism function (Baron and Zambryski 1995; Boller and He 2009; Pieterse et al. 2014). For example, inactivation of *Required for Arbuscular Mycorrhization (RAM)2* expression increases the resistance of the legume *Medicago truncatula* to pathogenic oomycetes but also blocks colonization by beneficial mycorrhizal fungi (Wang et al. 2012), and increased expression of *Pleckstrin Homology* (*PH*)*1* increases the resistance of soybean to oomycete leaf pathogens but also has systemic effects that reduce root nodule formation by nitrogen‐fixing rhizobia (Helliwell et al. 2022). If genes involved in defense have dual roles in sanctions, activation of defenses could alter the plant's ability to sanction mutualists, either in a positive or negative direction (Figure 1A).

Here, we examined how pea plants ( *Pisum sativum* ) manage infection by rhizobial mutualists (*Rhizobia leguminosarum* bv. *viceae*) during attack by aphids ( *Acyrthosiphon pisum* ) that vector a pathogenic plant virus, Pea Enation Mosaic Virus (PEMV). We used one wild and one domesticated pea genotype to broadly sample diversity in plant traits, since domestication can alter both sanctions traits and plant defense against antagonists (Kiers, Hutton, and Denison 2007; Chen, Gols, and Benrey 2015; Porter and Sachs 2020; Soldan et al. 2021), and legume genotypes are known to possess variation in their responses to mutualists (Simonsen and Stinchcombe 2014b; Wendlandt et al. 2019). We also used one non‐nodulating pea mutant to detect nodule‐independent effects of rhizobia on tolerance to aphid and PEMV antagonists. We inoculated peas with a beneficial nitrogen‐fixing rhizobial strain and an isogenic *dctA* mutant, which forms nodules but fails to fix nitrogen. These Fix+ and Fix− strains were labeled with fluorescent markers so that the strains occupying each nodule could be detected through fluorescent imaging. We performed a greenhouse experiment in which pea seedlings were grown with 1‐strain inocula (single inoculations) and 2‐strain inocula (co‐inoculations) of fluorescently labeled Fix+ and Fix− rhizobia for 4 weeks, attacked by aphids with or without PEMV for 72 h, and harvested 2 weeks later (Figure 1B). We quantified sanctions exerted by the pea plant over rhizobia in nodules using image analysis, and we measured several indices of plant performance. We asked: Do peas use host sanctions to manage infection by strains of rhizobia that differ in benefit (Q1)? Do peas plastically alter host sanctions in response to attack by sap‐feeding aphids (Q2a) or plant accumulation of an aphid‐vectored virus, PEMV (Q2b)? Do pea genotypes differ in their responses to mutualists or antagonists, and in how these responses interact (Q3)? As we try to simultaneously optimize symbiotic function and defense against antagonists in our crops, information about tradeoffs or pleiotropic effects among such traits (Figure 1C) will be useful for making informed decisions in crop breeding.

## 2 Methods

### 2.1 Fix+ and Fix− Rhizobia Strains

To evaluate plant management of mutualists, we exposed pea plants to isogenic *Rhizobium* strains that fix nitrogen (Fix +) or are unable to do so (Fix−). *Rhizobium leguminosarum* bv. *viciae* 3841 (Rlv 3841) is a field‐isolated strain that forms nitrogen‐fixing nodules on pea (Johnston and Beringer 1975). Strain Rlv 3841 was subjected to insertional mutagenesis using an omega interposon targeted to *dctA*, which encodes a dicarboxylate carrier protein, to generate isogenic strain RU 727 (Reid, Walshaw, and Poole 1996). Rhizobia that lack *dctA* expression do not fix nitrogen because they cannot import dicarboxylates, which are a major carbon source for rhizobia inside nodules and power nitrogen fixation (Udvardi and Poole 2013). However, *dctA* mutants can acquire alternate sources of carbon from the plant that are sufficient to allow them to multiply and respire within plant cells in the nodule (Reid, Walshaw, and Poole 1996) at viable cell densities comparable to those in nodules containing a wild‐type strain. *Rhizobium leguminosarum* does not require *dctA* for fitness in the rhizosphere or rhizoplane of pea (Wheatley et al. 2020). However, *dctA* is expressed when rhizobia are free‐living in the soil environment (Yurgel and Kahn 2004), *dctA* mutants have lower chemotaxis to succinate, one of the dicarboxylates transported by the *dctA* gene product (Robinson and Bauer 1993), and some soil microbes, like *Pseudomonas chloroaphis*, require *dctA* to colonize the rhizoplane (Nam et al. 2006). We refer to Rlv 3841 as the “Fix+ strain” and RU 727 as the “Fix− strain” to reflect their key phenotype for the purpose of this experiment.

Some legumes can exert pre‐infection partner choice, in which they preferentially initiate symbiosis with more beneficial strains, resulting in a greater number of nodules formed with superior mutualists (Heath and Tiffin 2009; Gubry‐Rangin, Garcia, and Bena 2010; Montoya et al. 2023). However, pea does not appear to exert partner choice when exposed to isogenic strains, such as the Fix+ and Fix− strains we used here, likely because genetic signals of quality at the point of symbiosis initiation are identical for isogenic strains (Westhoek et al. 2017, 2021; Younginger and Friesen 2019). Thus, we focus our analysis of host management of symbionts on host sanctions, which occur after strains have formed nodules on the host and symbiotic resource exchange has commenced.

### 2.2 Fluorescent Labeling of Rhizobia

To measure the symbiotic performance of isogenic nitrogen fixing (Fix+) and non‐fixing (Fix−) rhizobia on hosts, we generated strains expressing contrasting fluorescent proteins following Montoya et al. (2023). We performed biparental mating between *Rhizobium* strains (Fix+ and Fix−) and an *E. coli* MFDpir donor strain auxotrophic for diaminopimelic acid (DAP). The donor strain bore a plasmid conferring neomycin (Neo) resistance and either red fluorescence (mScarlet‐l fluorophore; Bindels et al. 2017) or green fluorescence (mEGFP fluorophore; Zacharias et al. 2002). The donor strain and plasmids conferring these traits (pAB138 and pAB146, respectively) were donated by Joel Griffitts (Brigham Young University). We suspended cells from each donor and recipient in 100 μL liquid media (Luria‐Bertani [LB] + DAP for the donor; modified arabinose gluconate [MAG] for the recipient), combined the cultures, pipetted a drop of the mixture onto MAG + DAP agar, and incubated at 28°C for 6 h. Cells were streaked on MAG + Neo agar to select for transconjugant *Rhizobium* cells and incubated at 28°C until colonies formed. Single colonies were streaked again onto MAG + Neo agar. We verified that each transconjugant showed the expected fluorescence using a Leica M165 FC Fluorescent Stereo Microscope and filters “Texas Red” (excitation 560 nm, emission 610 nm long pass) for mScarlet‐l and “ETblue” (excitation 470 nm, emission 515 nm long pass) for mEGFP. We cryopreserved one green and one red derivative of each *Rhizobium* strain, for four strains total (red Fix+, green Fix+, red Fix−, and green Fix−).

### 2.3 Pea Germplasm

To evaluate whether plant responses to mutualists and antagonists differ between two contrasting plant host genotypes, we examined a common crop cultivar, a non‐nodulating mutant of a different crop cultivar, and a wild pea accession. Plant genotypes were selected haphazardly to broadly sample from the genetic variation in this species. The domesticated pea accession (“Aragorn,” PI 648006) was developed in New Zealand, and the wild pea accession (*P. sativum elatius*, W6 15004) was originally collected in Ukraine.

The non‐nodulating pea (“Frisson P56”) was generated from the domesticated “Frisson” cultivar by EMS mutagenesis, which introduced a premature stop codon to *PsSym10* (Duc and Messager 1989; Sagan, Messager, and Duc 1993). Frisson P56 is deficient in nod factor perception, the earliest stage of symbiosis with rhizobia (Walker, Viprey, and Downie 2000). We were not able to obtain wild‐type (nodulating) Frisson pea germplasm, which would have allowed us to precisely isolate the effect of nodule formation on this accession's responses to mutualists and antagonists. The non‐nodulating pea accession was only used to test Q3 (i.e., whether pea accessions genotypically vary in their responses to mutualists and antagonists), since the other questions presuppose the ability to form nodules.

### 2.4 Plant Growth Conditions

Pea seeds were scarified, sterilized with chlorine gas for 4 h, and sown in autoclaved tissue culture boxes containing a mix of sand and potting soil on 8 December 2020. Each tissue culture box was nested into a second box containing water, with a wick connecting the boxes (Heath, Stock, and Stinchcombe 2010). The lower box was wrapped with aluminum foil to discourage algal growth and refilled with deionized water as needed. Plants were raised in the greenhouse at Washington State University, Vancouver (Vancouver, WA, USA). After germination, boxes were divided into six blocks based on initial plant size. Eleven boxes without germinants were resown on 21 December 2020. We added a pole to each box to allow plants to climb, and we fertilized each plant with a low‐nitrogen fertilizer (3 mL 250 μM ammonium nitrate [7 ppm nitrogen] in 0.5× Fahraeus solution) on 7 January 2021. We measured plant health non‐destructively twice during the experiment (15 and 29 January 2021, before and after aphid exposure, respectively) using a SPAD (soil plant analysis development) meter, which measures the difference in leaf transmittance of red (650 nm) and infrared (940 nm) light. SPAD readings approximate chlorophyll concentration in leaves (Uddling et al. 2007; Xiong et al. 2015), which increases with nitrogen fixation (Parra‐Colmenares and Kahn 2005).

### 2.5 Rhizobium Inoculation

To generate inocula, we grew rhizobia strains on MAG + Neo agar, transferred cells to liquid MAG + Neo, measured OD_600_, and estimated cell titer as OD_600_ × 5.8 × 10^7^ = CFU mL^−1^. We diluted cell suspensions to 10^7^ CFU mL^−1^ with water and combined equal volumes of cells to create the following four inocula: (a) Fix+ (half red Fix+, half green Fix+), (b) Fix− (half red Fix−, half green Fix−), (c) 2‐strain (co‐inoculation) version A (half red Fix+, half green Fix−), (d) 2‐strain (co‐inoculation) version B (half green Fix+, half red Fix−). Therefore, each inoculation treatment was intended to produce both red and green nodules on plants. Using both red‐ and green‐labeled strains in the 1‐strain treatments (i.e., Fix+ and Fix− treatments) prevented our measurements of nodule count and size from being biased by intrinsic differences in the detection of the red and green labels (i.e., marker effects). Similarly, using reciprocally labeled pairs of strains for the 2‐strain treatments (i.e., co‐inoculating some replicate plants with a mix of red Fix+ and green Fix− and co‐inoculating others with a mix of green Fix+ and red Fix− strains) allowed us to detect differences in nodule count and size based on the level of cooperation of each strain (i.e., its Fix+ or Fix− phenotype) without bias due to marker effects. We inoculated each plant with 900 μL cells (or a cell‐free control) on 21 December 2020 (2 weeks post sowing) by pipetting inoculum at the base of the plant. A thin layer of autoclaved sand was added to the top of each box to minimize cross‐contamination between inoculation treatments. We arranged plants in a randomized complete block design with treatments of pea accession (3 levels: wild, domesticated, and non‐nodulating), rhizobial inoculation (5 levels: Fix+, Fix−, co‐inoculation version A, co‐inoculation version B, and none), and aphid exposure (3 levels: no aphids, PEMV− aphids, and PEMV+ aphids, defined below). All treatments were replicated over six blocks, for a total of 270 plants (Figure 1B).

### 2.6 Aphid Treatment

After 6 weeks of growth, we exposed plants to one of three aphid treatments: (a) no aphids, (b) PEMV− (non‐viruliferous) aphids, or (c) PEMV+ (viruliferous) aphids. Aphid colonies were sourced from the lab of David Crowder (Washington State University), which has performed experiments on these aphids and pea plants and established that they are compatible with each other (Basu et al. 2021). PEMV+ aphid colonies were reared on pea plants infected with PEMV, and PEMV− aphid colonies were derived by rearing PEMV+ aphids on pea plants free of PEMV (Basu et al. 2021; Basu et al. 2022). We applied a greater number of PEMV+ aphids (7 aphids per plant) than PEMV− aphids (5 aphids per plant) to improve the chance of virus transmission to the host plant in the PEMV+ aphid treatment. For treatments using aphids, 5‐day old flightless aphids were applied to the apical shoot of plants on 19 January 2021 and secured by enclosing them in a mesh bag tightened around the plant stem. For the “no aphid” treatment, we covered plant apical shoots with empty mesh bags. Aphids were removed after 72 h.

### 2.7 Plant Harvest

We harvested experimental blocks sequentially between 1 and 4 February 2021 (8 weeks post sowing) by cutting plant shoots at soil level and recording shoot fresh mass. To measure PEMV titer in plants exposed to PEMV+ aphids, apical shoot samples were removed with clean scissors, wrapped in foil, frozen on dry ice before storage at −80°C. Tissue was ground in sterile conditions using a mortar and pestle and liquid nitrogen prior to RNA extraction, cDNA synthesis, and PCR. We followed established protocols that use PCR primers for the PEMV coat protein to determine PEMV titer from infected plants using rtPCR (Table S1; Basu et al. 2021, 2022). Gel electrophoresis band intensity was quantified from gel images using ImageJ (US NIH, Bethesda, MD, USA). At harvest, roots were washed clean of soil, stored at 4°C, and imaged using a Leica M165 FC Fluorescent Stereo Microscope (8 February to 15 March 2021). Because of field of view restrictions, we took many images per root system and reconstructed these into mosaic images (see Section 2.9). To verify that nodules showing both red and green fluorescence result from cases where both red and green fluorescent strains co‐infected the same nodule, we used a Leica SP8 laser‐scanning confocal microscope to image longitudinal sections of eight mixed‐color fluorescent nodules.

### 2.8 Culturing Nodules

To test whether nodule size was associated with the number of viable rhizobia inside nodules, we cultured rhizobia from four nodules per nodulated plant in one block (4 nodules × 24 plants = 96 nodules). On 1 to 4 March 2021, nodules were removed from roots and surface‐sterilized, crushed in water, serially diluted, and drop‐plated to generate single colonies, from which we estimated colony‐forming units (CFU) in each nodule (see Methods S1). Nodule size was measured from photographs by manually tracing the nodule perimeter and calculating area in ImageJ. For the 65 nodules that generated a CFU estimate, nodule size was positively correlated with logCFU per nodule (Figure S1; *r* = 0.37, *p* = 0.0024).

### 2.9 Digital Image Analysis of Nodules

**FIGURE 2:**
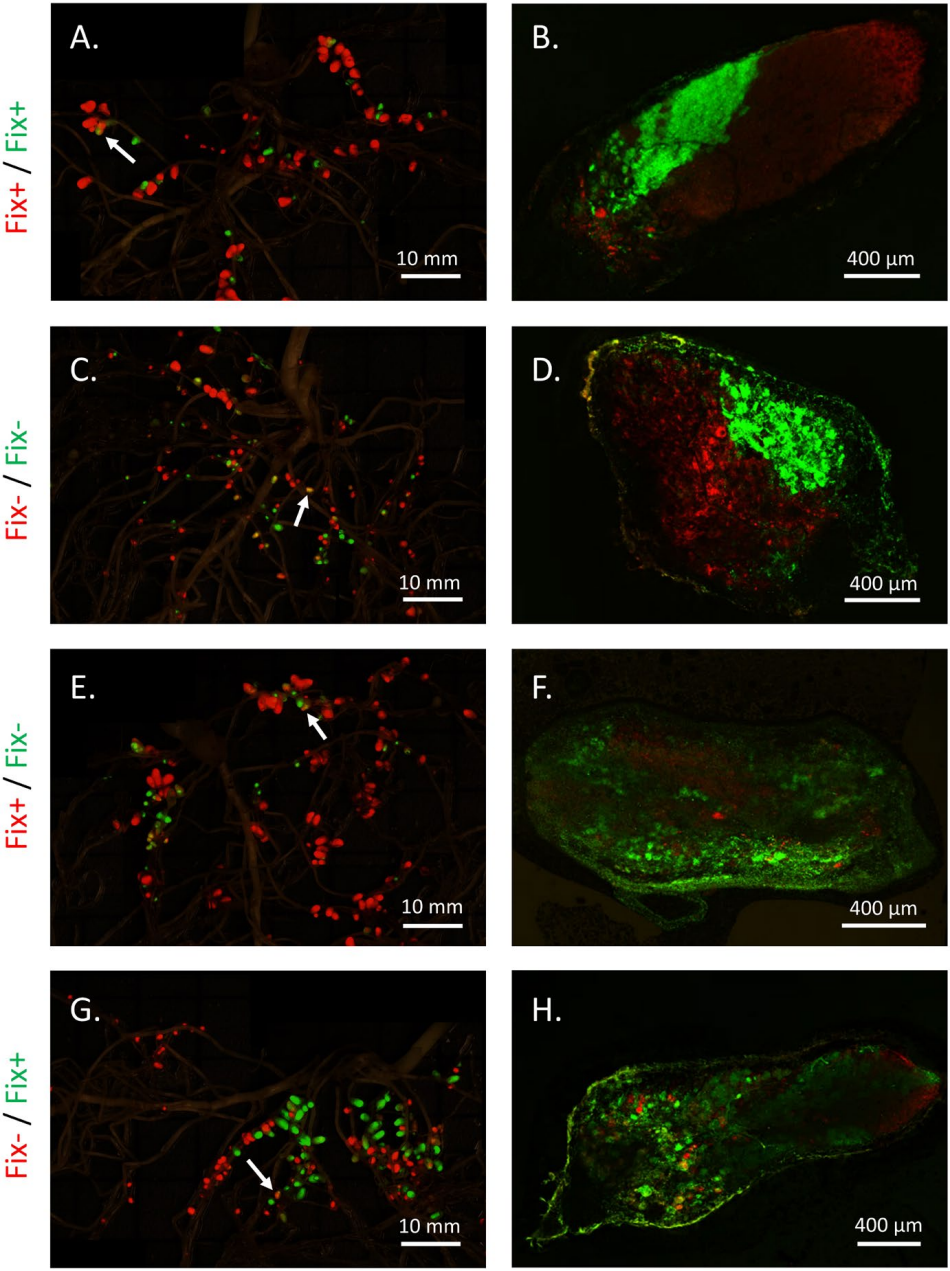
Fluorescent markers enable detection of nodules founded by Fix+, Fix−, or both rhizobia strains on pea plants. Plants were inoculated with red and green Fix+ rhizobia (A and B), red and green Fix− rhizobia (C and D), red Fix+ and green Fix− rhizobia (E and F), or red Fix− and green Fix+ rhizobia (G and H). Left‐hand panels (A, C, E, G) show mosaic images consisting of red fluorescence, green fluorescence, and darkfield layers. White arrows indicate examples of nodules showing both red and green fluorescence (i.e., mixed‐color nodules). Right‐hand panels (B, D, F, H) show confocal images of longitudinal sections of mixed‐color nodules. All panels show domesticated pea (see Figure S2 for wild pea). For mosaic images, mean pixel brightness was approximately 2‐fold greater for the red vs. green layer. To improve visibility of green nodules in the mosaic images, we reduced the pixel display range to 0–50 for the green layer, leaving the red layer at the default range of 0–255. Data analysis used unaltered images.

We used automated digital image analysis to quantify the number, size, and strain occupancy of root nodules. For each nodulated plant root system (*n* = 142), we manually overlapped raw images to generate a single mosaic image with red, green, and darkfield layers (Figure 2, Figure S2). We developed a custom ImageJ macro to calculate, for each nodule, total size (cross‐sectional area) and percentage of the nodule occupied by a green‐ or red‐labeled strain. We use the cross‐sectional nodule area occupied by a strain as the basis of estimating the resources acquired by each strain in a nodule (and for measuring host sanctions, see Section 2.11). Although a single nodule cross‐section can provide only a rough estimate of the three‐dimensional area occupied by strains in a nodule, a recent study using the same Fix+ and Fix− strains found a positive correlation between cross‐sectional nodule area occupied by a strain within a mixed‐infection nodule and its CFU count within that same nodule (Millar et al., in prep). Our ImageJ macro initially detected 23,283 possible nodules (i.e., “features”) across the entire dataset. We filtered out 2,101 erroneous features (holes within nodules), 6,864 features below a minimum nodule size threshold for a nodule, and 59 features above a maximum nodule size threshold. Minimum and maximum nodule size thresholds were set based on visually identifying the smallest and largest real nodules in the dataset. This yielded 14,259 putative nodules across all treatments.

We compared results of the automated digital image analysis to manual nodule counts for each plant, and these values were positively correlated across all 142 nodulated root systems (*r* = 0.94, *p* < 0.0001, Figure S3A). We manually estimated nodule size for 100 randomly selected nodules by hand‐tracing nodule outlines in the digital mosaic images. Automated estimates of nodule size were positively correlated with manually estimated nodule size (*r* = 0.53, *p* < 0.0001; Figure S3B), although the ImageJ macro tended to estimate a smaller nodule size than manual estimates (see Methods S2 for more details). During manual nodule counts, we observed that 73% of plants had some non‐fluorescent nodules. Since non‐fluorescent nodules represented only a median of 4% of each plant's total nodules, we did not further consider them. We attempted to culture rhizobia from 14 non‐fluorescent nodules, but they universally failed to grow, suggesting that non‐fluorescent nodules were senescent.

### 2.10 Mixed‐Color Nodules

Mixed‐color nodules (i.e., nodules showing both red and green fluorescence) accounted for 15% of all nodules in each inoculation treatment. CFU data from cultured nodules showed that mixed‐color nodules (*n* = 26) produced both red and green colonies, whereas single‐color nodules (*n* = 33) produced colonies of only a single color. Confocal microscopy showed that red‐ and green‐labeled strains in mixed‐color nodules either formed spatially distinct sectors or small, intermingled patches of infection within the nodule (Figure 2, Figure S2). Mixed‐color nodules were estimated to be larger than single‐color nodules on average, even in the Fix+ and Fix− inoculation treatments where the inoculated strains differed only in the color of the fluorescent marker. We suspect this may be artifactual, since adjacent single‐color nodules could be interpreted by our digital analysis pipeline as a single large mixed‐color nodule if their edges touched in the root image (Figure S4). As described below (Section 2.11), this size difference would not bias our measurements of sanctions because we never compared mixed‐color to single‐color nodules. Henceforth, “mixed‐infection nodules” indicate nodules occupied by both the Fix+ and Fix− strains, and “Fix+ nodules” or “Fix− nodules” indicate nodules occupied solely by the Fix+ or Fix− strain, respectively.

### 2.11 Measuring Host Sanctions

Host sanctions occur when plants invest more resources into symbiosis with more‐cooperative strains and/or degrade symbiotic structures containing less‐cooperative strains. Since nodule tissue results from carbon allocation by the plant, larger nodules indicate greater investment of resources by the host plant (Westhoek et al. 2021). In plants co‐inoculated with both Fix+ and Fix− strains simultaneously, root nodules could be either occupied by a single strain (i.e., Fix+ or Fix−) or occupied by both strains, forming a mixed‐infection nodule. We measured sanctions separately for single‐infection and mixed‐infection nodules. For single‐infection nodules, we inferred that plants were *sanctioning among nodules* if Fix+ nodules had greater total cross‐sectional areas than Fix− nodules. For mixed‐infection nodules, we inferred that plants were *sanctioning within nodules* if the Fix+ strain occupied a greater cross‐sectional area than the Fix− strain within the same nodule. The cross‐sectional area occupied by each strain in a mixed‐infection nodule was referred to as the strain's “partial nodule area.” We assumed that each strain's partial nodule area was proportional to the amount of host resources directed toward that strain in the nodule, just as total nodule area is proportional to host resources allocated to the entire nodule.

The above criteria for detecting sanctions refers to a binary response by the plant (i.e., a plant can be either sanctioning or not sanctioning). To capture quantitative variation in the amount of sanctioning happening across treatments, we also calculated the strictness of sanctions for individual co‐inoculated plants as follows:

$$\text{Strictness of sanctions}=\ln\left(\frac{Z_{{\mathrm{Fix}}^{+}}}{Z_{{\mathrm{Fix}}^{-}}}\right)$$

Where *Z* represents the mean total nodule area of single‐infection nodules on a plant, this equation estimates the strictness of among‐nodule sanctions. Where *Z* represents mean partial nodule area in mixed‐infection nodules on a plant, this equation estimates the strictness of within‐nodule sanctions. Higher positive values for strictness indicate greater degrees of sanctions, as plants increasingly direct nodule resources to Fix+ rather than Fix− strains. Strictness values less than or equal to zero indicate the plant is not favoring the Fix+ strain at all (i.e., not sanctioning).

### 2.12 Data Analysis

We analyzed data using generalized linear mixed models implemented with *lme4* v. 1.1–29 (Bates et al. 2015) in R v. 4.1.2 (R Core Team 2021). Models used Gaussian errors and residuals were checked with *DHARMa* v. 0.4.5 (Hartig 2022). We used likelihood ratio tests to determine the significance of fixed effects, using an *α* of 0.05. Specifically, we used the drop1 function in the base R package *stats* to assess the significance of the highest‐order interaction term in a model; to test the significance of each lower‐order term, we created a reduced model by removing all higher‐order interactions that included the term of interest before using the drop1 function on the reduced model. For significant terms, we performed multiple comparisons among treatment levels using *emmeans* v. 1.7.5 (Lenth 2022), with a Holm *p*‐value correction for multiple comparisons.

To confirm that Fix+ and Fix− rhizobia provide different benefits to pea plants, we modeled three metrics of plant performance (before‐aphid SPAD, after‐aphid SPAD, and fresh shoot mass at harvest) with fixed effects of Accession (wild, domesticated, and non‐nodulating pea), Inoculum (None, Fix−, Fix+, and 2‐strain), Aphid treatment (None, PEMV−, and PEMV+), all interactions among fixed effects, and a random effect of Block (Table S2). We did not test the Aphid terms for before‐aphid SPAD, since this dataset was collected before the aphid treatment was administered. Since the presence of rhizobia can increase a plant's resistance to viruses (Basu et al. 2021), we also tested whether different rhizobial inocula offered different levels of protection against the aphid‐vectored virus, PEMV. For plants exposed to PEMV+ aphids, we modeled PEMV titer with fixed effects of Accession, Inoculum, their interaction, and a random effect of Block (Table S2).

The resources the Fix− strain acquires (i.e., the size of the nodules it forms) can be affected by 1) its own ability to take up plant resources and 2) the plant divesting resources from Fix− nodules via sanctions. We assume that the Fix− strain's ability to take up plant resources is similar in 1‐strain and 2‐strain inoculations. Thus, if a Fix− nodule is smaller in 2‐strain compared to 1‐strain inoculations, this suggests that the plant is exerting sanctions during 2‐strain inoculations, when it has a choice among symbiotic partner genotypes (Westhoek et al. 2021). In contrast, if the plant were not sanctioning, we would expect the Fix− strain to have similar nodule size in 2‐strain and 1‐strain inoculations. We tested the ability of pea plants to exert sanctions with our pair of rhizobia strains (Q1) by analyzing the total area of singly‐infected nodules from plants exposed to 1‐strain versus 2‐strain inocula. We modeled total nodule area with fixed effects of Accession (wild or domesticated), Inoculum (1‐strain or 2‐strain), Occupant of the nodule (Fix+ or Fix−), Aphid treatment, all fixed‐effect interactions, and random effects of Block and Plant (Table S3). We tested the Inoculum × Occupant interaction to understand if the size of Fix+ and Fix− nodules differed between plants exposed to 1‐strain and 2‐strain inocula (Q1).

After determining that plants had the ability to exert sanctions during 2‐strain inoculations, we tested whether plant ability to sanction rhizobia differed among aphid treatments (Q2a). For plants in 2‐strain inoculations, we analyzed total nodule area of single‐infection nodules and partial nodule area of mixed‐infection nodules. We analyzed these two response datasets with fixed effects of Occupant of the nodule (Fix+ or Fix−), Accession, Aphid treatment, and all interactions among fixed effects (Table S4). We included Fix+ marker color as a covariate to control for differences in detection of the red and green label in the reciprocally labeled 2‐strain inoculation treatments. We also tested random effects of Block, Plant, and Nodule (nodule was only tested in the partial nodule area model, since this dataset included two measurements per nodule). Since we were interested in identifying the treatments in which plants showed sanctions, we tested for significant contrasts between Fix+ and Fix− occupants for any significant effects of Occupant, including interactions between Occupant and other terms.

To test if the strictness of host sanctions was associated with plant susceptibility to PEMV (Q2b), we modeled PEMV titer of plants exposed to PEMV+ aphids (*n* = 20) with fixed effects of sanctions strictness (testing both among‐nodule sanctions strictness and within‐nodule sanctions strictness), Accession, their interaction, and a random effect of Block (Table S5). We included Fix+ marker color as a covariate to control for differences in detection of the red and green label. Although we tested PEMV titer as a function of sanctions strictness, we acknowledge that we do not know the true direction of causality in this relationship, since sanctions and PEMV titer were both responses that we measured, rather than treatments we imposed.

To test whether the wild and domesticated pea accessions differed in their responses to mutualists or antagonists (Q3), we evaluated the significance of the Accession term (or any interaction terms that included Accession) in our previous statistical models (Table S2–S4). We also examined the same terms in models of additional rhizobial symbiosis traits (nodule count per plant, mean individual nodule size per plant, and rhizobial CFU per nodule), which included fixed effects of Accession, Inoculum, Aphid treatment, all interactions, and random effects of Block and Plant as appropriate (Table S6).

## 3 Results

### 3.1 Plants Benefit From Fix+ Rhizobia but Not Fix− Rhizobia

**FIGURE 3:**
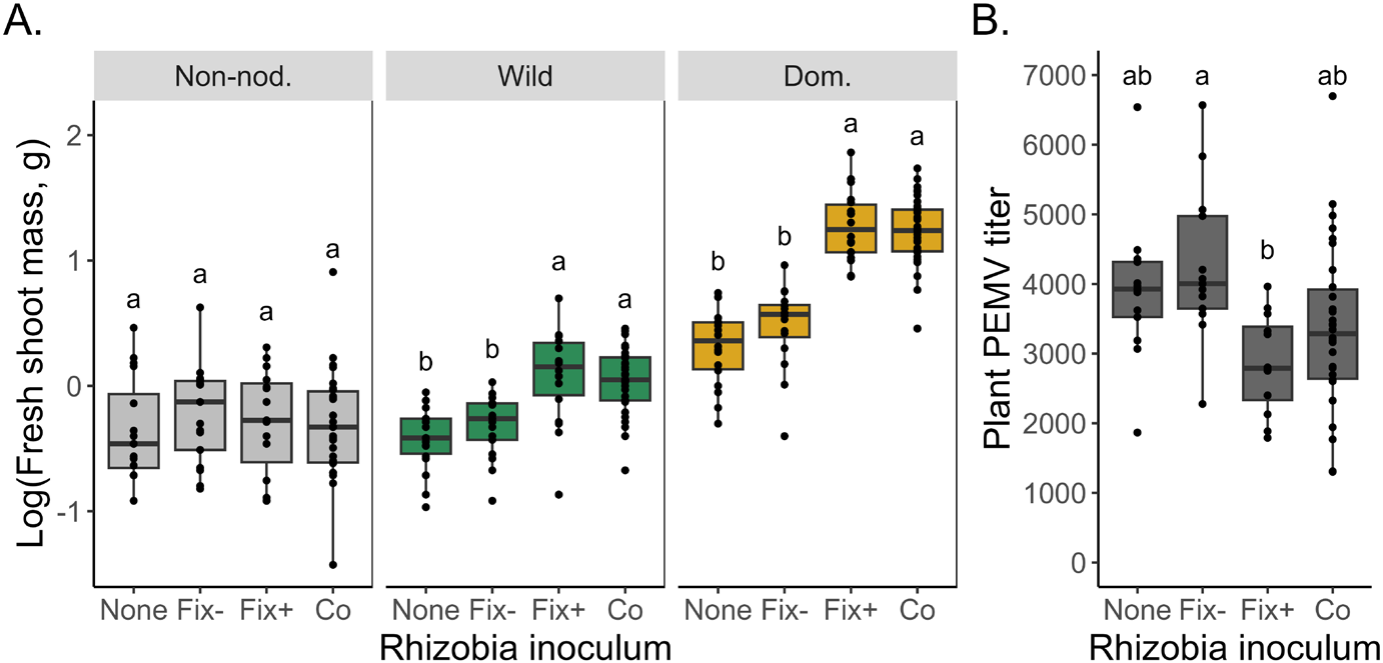
Plants benefit from Fix+ but not Fix− rhizobia. (A) The wild and domesticated pea accessions had greater shoot mass when inoculated with Fix+ rhizobia than when inoculated with Fix− rhizobia; the non‐nodulating pea accession had similar low shoot mass with each inoculum (Accession × Inoculum *χ* ^2^(6) = 75.3, *n* = 255 plants, *p* = 3.38e‐14; see Table S2). Shoot mass did not vary across aphid treatments. Different letters indicate significantly different means among inoculation treatments within each pea accession. Note that shoot mass data were log‐transformed for both analysis and plotting. (B) For plants exposed to PEMV+ aphids, inoculation with Fix+ rhizobia reduced PEMV accumulation by the plant compared to inoculation with Fix− rhizobia (Inoculum *χ* ^2^(3) = 12.2, *n* = 68 plants, *p* = 0.00678; see Table S2). Plant PEMV titer did not vary across the three tested accessions (non‐nodulating, wild, and domesticated pea). Different letters indicate significantly different means among inoculation treatments, pooling together all pea accessions. For both (A) and (B), each dot shows data from a single plant, the box shows the treatment median ± 1 quartile, and the whiskers encompass all datapoints within 1.5 times the interquartile range of the box. “Co” refers to the co‐inoculation treatment (i.e., 2‐strain inoculation), which had twice the replication of the other treatments due to pooling the reciprocally labeled co‐inoculation treatments. Post hoc explorations used a significance threshold of *α* = 0.05 and a Holm *p‐*value correction for multiple comparisons.

Plants inoculated with the Fix+ strain had greater performance than plants inoculated with the Fix− strain, in terms of greater shoot mass, greater SPAD, and lower accumulation of PEMV when exposed to PEMV+ aphids. The wild and domesticated pea accessions successfully formed nodules with both the red‐ and green‐labeled versions of the Fix+ and Fix− strains (Figure 2, Figure S2). In 1‐strain inoculations, the Fix+ strain increased shoot mass and SPAD for both the wild and the domesticated pea relative to uninoculated control plants, whereas the Fix− strain did not enhance plant performance relative to controls (Figure 3A, Figure S5, Table S2). Non‐nodulating pea did not form nodules with either the Fix+ or Fix− strain, and non‐nodulating pea failed to benefit from inoculation with either strain relative to control inoculations (Figure 3A, Figure S5, Table S2). Together, these results are consistent with nodule formation driving the shoot mass and SPAD benefits provided by the Fix+ strain. Plants inoculated with Fix+ rhizobia also had lower PEMV titer than plants inoculated with Fix− rhizobia (Inoculum effect; Table S2, Figure 3B). The differential effect of Fix+ and Fix− rhizobia on plant PEMV accumulation occurred for all three pea accessions (the non‐nodulating, wild, and domesticated pea), suggesting the effect of this single locus (*dctA*) on plant PEMV titer is not related to nodule formation.

### 3.2 Plants Impose Sanctions on Rhizobia That Differ in Nitrogen Fixation (Q1)

**FIGURE 4:**
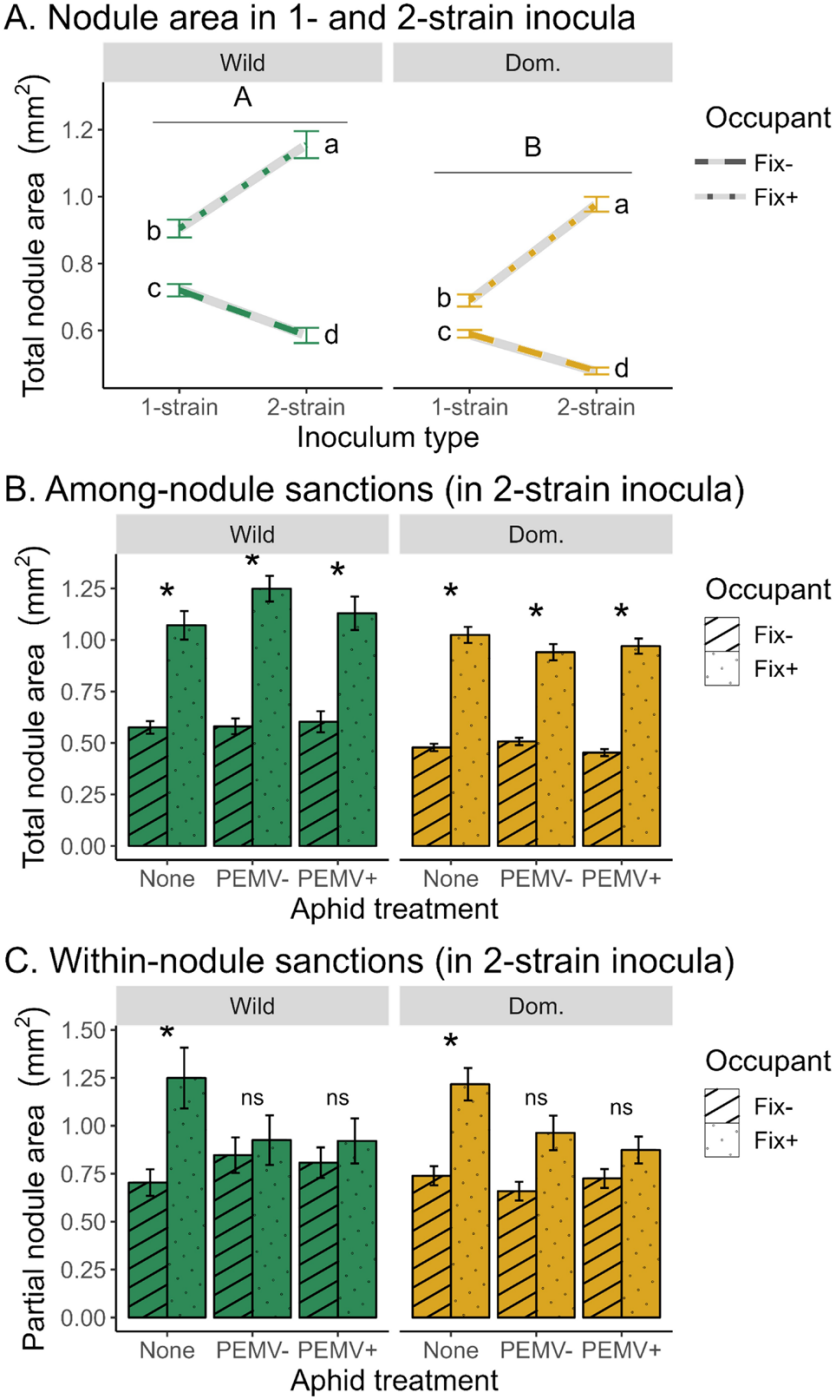
Within‐nodule sanctions weaken when plants are attacked by aphids, but among‐nodule sanctions persist. (A) Plants make larger Fix+ nodules and smaller Fix− nodules in 2‐strain inoculations than in 1‐strain inoculations, for both wild and domesticated pea (Inoculum type × Occupant *χ* ^2^(1) = 59.8, *n* = 13,219 nodules, *p* = 1.06e‐14; see Table S3). Different letters indicate significantly different mean nodule size. (B) In 2‐strain inoculations, the wild and domesticated pea show among‐nodule sanctions (larger Fix+ nodules than Fix− nodules) in each aphid treatment (Occupant × Accession × Aphid *χ* ^2^(2) = 18.0, *n* = 5,740 nodules from 72 plants, *p* = 1.24e‐4; see Table S4). Nodules from the no‐aphid, PEMV− aphid, and PEMV+ aphid treatments totaled 1,823, 1,956, and 1,961, respectively. (C) In 2‐strain inoculations, wild and domesticated pea show within‐nodule sanctions (Fix+ rhizobia occupy more space within mixed‐infection nodules than Fix− rhizobia) when plants are not exposed to aphids, but plants fail to show within‐nodule sanctions when attacked by PEMV− or PEMV+ aphids (Aphid × Occupant *χ* ^2^(2) = 14.7, *n* = 1,040 nodules from 72 plants, *p* = 6.52e‐4; see Table S4). Nodules from the no‐aphid, PEMV− aphid, and PEMV+ aphid treatments totaled 343, 322, and 375, respectively. Asterisks indicate significant differences in nodule area occupied by Fix+ versus Fix− rhizobia, and “ns” indicates no significant difference between Fix+ and Fix− occupants. In all panels, we used data from all nodules individually, but statistical analyses included “Plant” as a random effect to control for multiple nodules collected from the same plant (see Tables S3 and S4). All post hoc explorations used a significance threshold of *α* = 0.05 and a Holm *p‐*value correction for multiple comparisons. Bars show mean +/− 1 SE.

Plant sanctions were evident in 1‐strain inoculations, but increased in intensity in 2‐strain inoculations, when plants had a choice among symbiotic partner genotypes. In 1‐strain inoculations, Fix+ nodules were 21% larger than Fix− nodules, consistent with plants allocating more resources to nodules that provided a performance benefit (Figure 4A). In 2‐strain inoculations, the size difference between Fix+ and Fix− nodules increased further (Inoculum × Occupant effect, Table S3, Figure 4A), with Fix+ nodules growing 35% larger and Fix− nodules growing 20% smaller than they did in 1‐strain inoculations (Figure 4A). Thus, when plants had a choice among symbiotic partner genotypes, they increased their allocation to beneficial symbionts and decreased their allocation to non‐beneficial symbionts.

In 2‐strain inoculations, plant sanctions occurred both among singly‐infected nodules and within mixed‐infection nodules. For singly‐infected nodules on co‐inoculated plants, Fix+ nodules grew 103% larger than Fix− nodules (Occupant effect, Table S4, Figure 4B). This size difference varied across pea accessions and aphid treatments (significant Accession × Occupant × Aphid effect; Table S4), but not qualitatively: Fix+ nodules were significantly larger than Fix− nodules in all accession × aphid treatment combinations. For mixed‐infection nodules on co‐inoculated plants, the Fix+ strain occupied 39% more area within mixed‐infection nodules than the Fix− strain, but only when plants were not exposed to aphids (Occupant × Aphid effect, Table S4, Figure 4C). Overall, these results are consistent with plants having capacity for strong among‐nodule and within‐nodule sanctions.

### 3.3 Plant Responses to Antagonists Compromise Within‐Nodule Sanctions (Q2)

**FIGURE 5:**
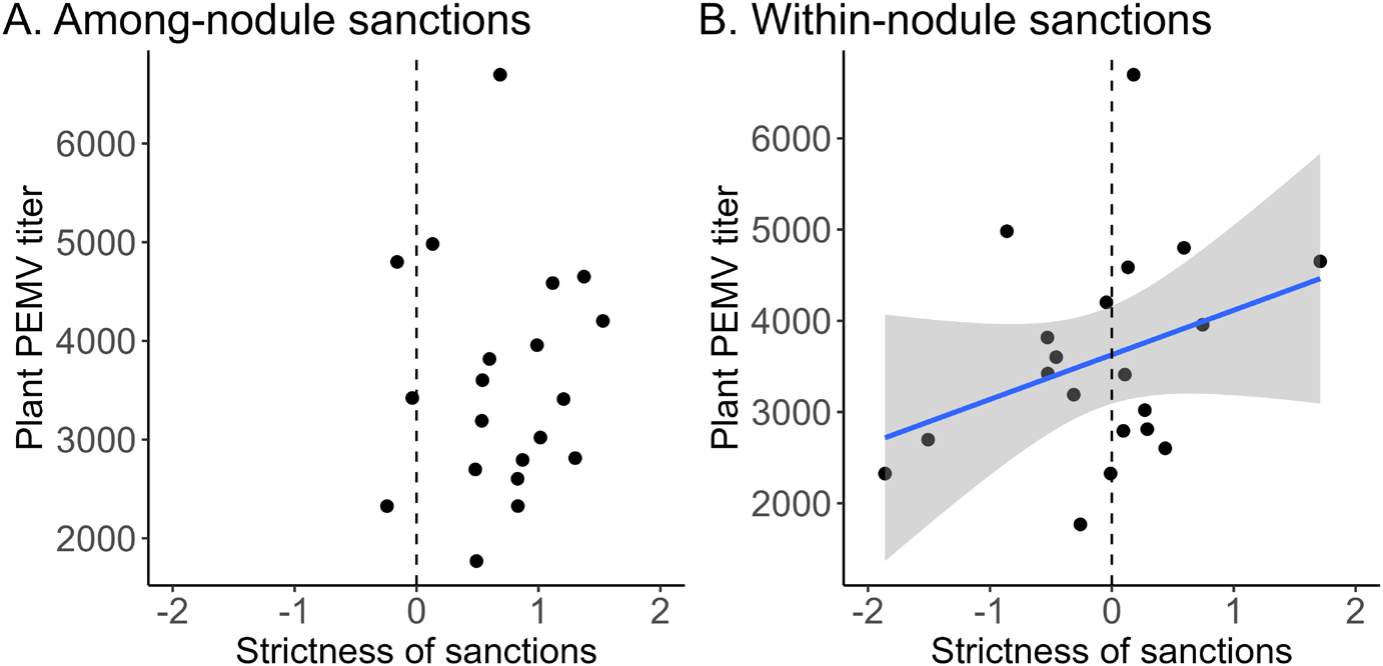
Susceptibility to PEMV increases with the strictness of within‐nodule sanctions, but is unrelated to the strictness of among‐nodule sanctions. For each plant exposed to PEMV+ aphids, we calculated strictness of sanctions for (A) among‐nodule sanctions as ln (Fix+ total nodule area/Fix− total nodule area) and (B) within‐nodule sanctions as ln(Fix+ partial nodule area/Fix− partial nodule area). (A) Plant PEMV titer did not vary with strictness of among‐nodule sanctions (*χ* ^2^(1) = 0.649, *n* = 20 plants, *p* = 0.420; see Table S5). (B) Plant PEMV titer increased with the strictness of within‐nodule sanctions (*χ* ^2^(1) = 3.98, *n* = 20 plants, *p* = 0.0461; see Table S5). The blue line indicates the best fit slope and 95% confidence interval for the relationship between PEMV titer and the strictness of sanctions. For both (A and B), each point shows data from a single co‐inoculated plant exposed to PEMV+ aphids, and the vertical dashed line shows sanctions strictness of 0 (i.e., no sanctions). Although we plot PEMV titer as a function of sanctions strictness, we acknowledge that we do not know the true direction of causality in this relationship, since sanctions and PEMV titer were both responses that we measured, rather than treatments we imposed.

Exposure to aphid and response to PEMV antagonists compromised within‐nodule sanctions, but not among‐nodule sanctions, for plants co‐inoculated with Fix+ and Fix− rhizobia. Among‐nodule sanctions were robust in all aphid treatments: Fix+ nodules were significantly larger than Fix− nodules in all Accession × Aphid treatments, despite a significant Occupant × Accession × Aphid interaction that indicated quantitative differences in the magnitude of among‐nodule sanctions across aphid treatments (Table S4, Figure 4B). Furthermore, in plants attacked by PEMV+ aphids, the amount of virus a plant accumulated (PEMV titer) was not associated with the strictness of among‐nodule sanctions (Table S5, Figure 5A). In contrast, plants that were exposed to aphids failed to show within‐nodule sanctions, with Fix+ and Fix− strains occupying indistinguishable areas within mixed‐infection nodules (Occupant × Aphid effect, Table S4, Figure 4C). Plants attacked by PEMV+ aphids also had stricter within‐nodule sanctions if they accumulated higher PEMV titers (Table S5, Figure 5B). These findings are consistent with sanctions‐defense antagonism, specifically for within‐nodule sanctions but not among‐nodule sanctions.

### 3.4 Pea Accessions Differ in Some Responses to Mutualists and Antagonists (Q3)

The domesticated and wild pea accessions were broadly similar in how sanctions over rhizobia interacted with defense against antagonists, but they differed quantitatively in some traits. The domesticated pea accession formed more nodules and smaller nodules than the wild pea accession (Accession effect; Figure 4A, Table S3, Figure S6A,B, Table S6). Aphid treatment also affected nodule count differently for the wild and the domesticated pea (Accession × Inoculum × Aphid effect; Table S6, Figure S6A). When exposed to aphids, the domesticated pea accession formed more nodules with the Fix− treatment than with the Fix+ or 2‐strain inoculation treatments (Figure S6A). In contrast, when not exposed to aphids, the domesticated pea accession formed similar numbers of nodules in each treatment, and the wild pea accession formed similar numbers of nodules in all inoculation and aphid treatments (Figure S6A). We did not detect differences between the wild and domesticated pea accessions for CFU per nodule (Figure S6C, Table S6), PEMV titer (Table S2), or within‐nodule sanctions (Table S4).

## 4 Discussion

### 4.1 Tradeoff Between Antagonist Defense and Mutualist Sanctions

Our data illustrate that a common ecological scenario, attack by antagonists, may compromise a plant's ability to optimally impose sanctions on mutualists that differ in the benefit they provide their host. Broadly, this constraint could hinder efforts to breed crops for both high resistance to antagonists and optimal control over microbial mutualists. In our study, we raised pea plants in symbiosis with cooperative (Fix+) and uncooperative (Fix−) rhizobia for 4 weeks, exposed them to aphid attack with or without a viral pathogen for 72 h, and measured sanctions 2 weeks later (Figure 1B). Plants that were never exposed to aphids were able to sanction mixed‐infection root nodules, such that the Fix− strain occupied less space within these nodules than the Fix+ strain. However, we failed to detect sanctions within mixed‐infection nodules in plants exposed to viruliferous (PEMV+) or non‐viruliferous (PEMV−) aphids. Instead, Fix+ and Fix− strains occupied indistinguishable amounts of space within nodules (Figures 1C and 4C). Since aphid attack elicits a strong defense response from plants (Howe and Jander 2008; Jaouannet et al. 2014), activation of these plant defenses could be responsible for impairing sanctions of rhizobia within mixed‐infection nodules. Our finding that plants that imposed stricter sanctions in mixed‐infection nodules had lower resistance to PEMV (i.e., higher viral titer in leaf tissue; Figure 5B) is also consistent with a sanctions‐defense tradeoff in plants.

By weakening sanctions, plant interactions with antagonists could help maintain variation in cooperation among soil microbial mutualists. Sanctions preferentially reward more‐cooperative symbionts (Bull and Rice 1991; Daubech et al. 2017; Quides et al. 2017), and therefore sanctions are predicted to purge less‐cooperative symbionts from symbiont populations (West, Kiers, and Simms 2002). Yet less‐cooperative symbionts are common in empirical assessments of mutualisms, motivating research on how variation in symbiont quality is maintained in populations despite purifying selection due to sanctions (Heath and Stinchcombe 2013; Westhoek et al. 2017, 2021). Our results demonstrate that when plants defend against antagonists, sanctions can be weakened, which could create a refuge for less‐cooperative symbionts within mutualist populations. In agricultural systems, the persistence of sub‐optimal rhizobial symbionts has been known to reduce the yield of legume crops (Triplett and Sadowsky 1992). Our research suggests that future efforts to improve symbiotic outcomes in crops should be informed by biotic factors like plant disease prevalence and intensity.

During aphid attack, we observed host sanctions only within single‐infection nodules (Figure 4B), which could reflect either biological reality (i.e., no sanctions in mixed‐infection nodules) or methodological limits of our study. Mixed‐infection nodules occur when cells from more than one rhizobium strain are present in infection threads that initiate a nodule. They are common in pea and comprised 15% (SD = 8.2%) of the nodules in this experiment, which is similar to rates observed in other studies (Mendoza‐Suarez et al. 2020). It is possible that changes to within‐nodule sanctions could occur relatively quickly through targeted senescence of plant cells harboring the Fix− strain (Regus et al. 2017), and this could be readily detected by our methods which compare the area occupied by different strains inside of a nodule. In contrast, we did not detect changes to among‐nodule host sanctions in response to aphid attack (Figure 4B). Such changes could involve the plant altering the growth rates of existing Fix+ and Fix− nodules, which could be a relatively slow process compared to within‐nodule sanctions (Westhoek et al. 2021), and our methods may be less sensitive to detecting recent targeted senescence of plant cells harboring the Fix− strain within a nodule that contains only one strain. Thus, the 2‐week interval between aphid attack and when we measured sanctions may have been insufficient to detect changes in among‐nodule sanctions trigged by aphid attack, even though it was sufficient to detect changes in within‐nodule sanctions (Figure 1B). Performing experiments over longer plant growth periods may help discern whether antagonist attack is able to weaken among‐nodule sanctions in addition to within‐nodule sanctions.

### 4.2 Cooperative Rhizobia Improve Plant Resistance to a Viral Antagonist

Inoculation of pea plants with rhizobia can increase their resistance to PEMV (Basu et al. 2021). We found no general change in plant PEMV titer in response to rhizobial inoculation, but rhizobial strains differing in a single gene (*dctA*) had distinct effects on the outcome of a plant‐virus interaction. All three pea accessions in our study, including non‐nodulating pea, had greater resistance to PEMV when inoculated with Fix+ than with Fix− rhizobia (Figure 3B). It is unlikely that Fix+ rhizobia increased plant resistance to viruses through any nodulation signaling pathways, since the non‐nodulating pea genotype we examined is deficient in nod factor perception (Walker, Viprey, and Downie 2000). Instead, we hypothesize that Fix+ rhizobia increased plant resistance to PEMV by triggering induced systemic resistance (ISR) from the rhizoplane. Many beneficial soil microbes, including rhizobia, can trigger ISR or ISR‐like responses, which prime the plant immune system to react quickly to imminent pathogen attack (Pieterse et al. 2014; Tonelli et al. 2020). By simply colonizing the rhizoplane, Fix+ rhizobia may impact the host immune system and enhance plant defense against a virus, relative to plants colonized by Fix− isogenic mutants.

Our results suggest that the ability of rhizobia to impact plant defense against a virus is related to rhizobia possessing a functioning dicarboxylate transport system. *dctA* is required for the beneficial microbe *Pseudomonas chlororaphis* to trigger ISR in tobacco against the pathogen *Erwinia carotovora* (Nam et al. 2006), possibly because *dctA* allows the microbe to metabolize a greater number of host root exudates and reach higher densities on the plant root (Raaijmakers et al. 1995). If our Fix− (*dctA* mutant) strain had lower rates of survival or reproduction in the rhizosphere than the Fix+ strain, this could lead to the Fix+ but not the Fix− strain triggering ISR during 1‐strain inoculations. However, plants co‐inoculated with both Fix+ and Fix− rhizobia had intermediate PEMV titers (Figure 3B); one possibility is that resource competition between the two strains interfered with the ability of the Fix+ strain to trigger ISR. It would be valuable for future research to investigate the mechanism by which *dctA* in rhizobia modifies pea resistance to PEMV. If a functioning dicarboxylate transport system is necessary and sufficient to impact plant resistance to PEMV, we would expect that plants inoculated with a Fix− strain defective only in nitrogen fixation (i.e., a *nifH* mutant with functional *dctA*) would have similar PEMV titers to plants inoculated with a Fix+ strain.

### 4.3 Sanctions Are Similar in Two Pea Accessions

The wild and domesticated pea accessions were indistinguishable in their shared ability to sanction mutualists, both among singly‐infected nodules (Figure 4B) and within mixed‐infection nodules (Figure 4C). Legumes like pea form indeterminate nodules in which bacteroids are terminally differentiated, and it has remained an open question whether sanctions within mixed‐infection nodules are possible in such nodules. The sample size of nodules in our study (14,259) is much larger than that of previous work on this question, which may have allowed us to detect within‐nodule sanctions within indeterminate nodules for the first time. We also find that the wild and domesticated pea accessions experienced similar effects of antagonists on host sanctions (Figure 4C), suggesting that sanctions‐defense antagonism may be common across diverse pea genotypes. Although testing a single wild and domesticated pea accession is insufficient for testing the effect of domestication on pea responses to mutualists and antagonists, our findings are consistent with recent evidence for plasticity in symbiosis traits in a larger sample of wild and domesticated pea accessions (Millar, Piovia‐Scott, and Porter 2023). However, comparisons of wild and domesticated crop lineages in other plant taxa have revealed negative effects of domestication on sanctions (Kiers, Hutton, and Denison 2007), plant benefits from mutualism (Millar, Piovia‐Scott, and Porter 2023), and plant defense against antagonists (Chen, Gols, and Benrey 2015). Future research on larger numbers of wild and domesticated accessions will be required to strengthen inferences regarding the impact of domestication on how antagonists affect mutualist management in pea (Porter and Sachs 2020).

The single genotype of domesticated pea and its wild relative we examined did show genetic differences in some symbiosis traits. The domesticated pea accession generally formed more nodules and smaller nodules than did the wild pea accession (Figure S6A,B), and this difference was greatest when plants were exposed to the Fix− strain and aphids. Forming more nodules during biotic stress could be adaptive if increased symbiotic nitrogen fixation augments plant defense. Alternatively, forming more nodules could be maladaptive if nodules are primarily occupied by uncooperative strains, since these nodules represent plant investment with no possibility of a return.

## 5 Conclusions

Plant hosts can face tradeoffs as they respond to challenges from both antagonists and mutualists. We elucidate one such tradeoff by showing that plant defense against aphid and virus antagonists compromises the ability of pea plants to sanction rhizobia in root nodules co‐infected by two strains that differ in nitrogen‐fixing ability. The wild and domesticated pea genotypes we compared were similar both in their ability to sanction rhizobia and in the disruption of within‐nodule sanctions when antagonists attacked, which may indicate that this tradeoff is common in pea. In contrast, 85% of nodules contained only a single strain and sanctions among these nodules was not compromised by antagonist attack, indicating that while some aspects of sanctions were compromised, others were insensitive to antagonists. This pattern highlights a key advantage to hosts that produce singly‐infected nodules: sanctions among singly‐infected nodules appear to be more robust to disruption by antagonists than within‐nodule sanctions in mixed‐infection nodules. We also show that inoculating plants with rhizobia with functional DctA (i.e., the Fix+ strain) can increase plant resistance to an aphid‐vectored virus, PEMV, relative to rhizobia with non‐functional DctA (i.e., the Fix− strain). It is possible this effect occurs because the Fix+ strain more effectively triggers induced systemic resistance from the rhizosphere. Our work adds to growing evidence that plants face conflicting selection pressures from mutualists and antagonists (Hacquard et al. 2016; Wood et al. 2018; Costa, Ng, and Mathesius 2021; Chen et al. 2024), which could pose challenges to efforts to breed crops for both high resistance to antagonists and optimal interactions with mutualists. However, our work also highlights a potential opportunity: breeding crops for robust sanctions in the context of real‐world antagonists could target enhancement of among‐nodule sanctioning and the tendency to form singly‐infected nodules.

## Conflicts of Interest

The authors declare no conflicts of interest.

## Supporting information


Data S1.

## Data Availability

The data and R code associated with this manuscript have been archived in Dryad (DOI: 10.5061/dryad.w9ghx3g0m).
