# Supplementary material for: Managing Friends and Foes: Sanctioning Mutualists in Mixed‐Infection Nodules Trades off With Defense Against Antagonists
Source: Evol Appl. 2024 Dec 29;18(1):e70064. doi: 10.1111/eva.70064 (PMC11683190; doi:10.1111/eva.70064)
Supplement: Supplementary file 1 — Data S1. [file EVA-18-e70064-s001.docx]

**SUPPLEMENTARY INFORMATION** for “Managing friends and foes: Sanctioning mutualists in mixed-infection nodules trades off with defense against antagonists.”

**CONTENTS**:

- [**Fig S1**](#FigS1): Number of viable rhizobia in nodule increases with nodule size
- [**Fig S2**](#FigS2): Fluorescent markers enable detection of nodules founded by Fix+, Fix-, or both rhizobia strains on pea plants
- [**Fig S3**](#FigS3): Nodule traits estimated using ImageJ are positively correlated with traits measured manually from root images
- [**Fig S4**](#FigS4)**:** Examples of input fluorescence images and ImageJ output showing predicted nodule color and size
- [**Fig S5**](#FigS5): Wild and domesticated pea gain similar SPAD benefits from inoculation with Fix+ rhizobia
- [**Fig S6**](#FigS6). Wild pea forms fewer and larger nodules than domesticated pea, but accessions have similar rhizobial CFU per nodule
- [**Table S1**](#TableS1): PCR primers used to amplify PEMV
- [**Table S2**](#TableS3): GLMM statistics testing how plant benefits from rhizobia vary by pea accession, rhizobial inoculum, and aphid treatments
- [**Table S3**](#TableS4): GLMM statistics testing how the cross-sectional area of singly-infected nodules varies by pea accession, inoculum type, nodule occupant, and aphid treatment
- [**Table S4**](#TableS5): GLMM statistics testing how the cross-sectional area occupied by strains in single-infection nodules and mixed-infection nodules varies by nodule occupant, pea accession, and aphid treatment during 2-strain inoculations
- **Table S5**: GLMM statistics testing how plant PEMV titer varies based on the strictness of host sanctions and pea accession
- **Table S6**: GLMM statistics testing how nodule count, nodule cross-sectional area, and number of culturable rhizobia per nodule vary by accession, inoculum, and aphid treatments
- [**Methods S1**](#MethodsS1): Culturing rhizobia from nodules
- [**Methods S2**](#MethodsS2): Developing and checking ImageJ macro for estimating nodule traits


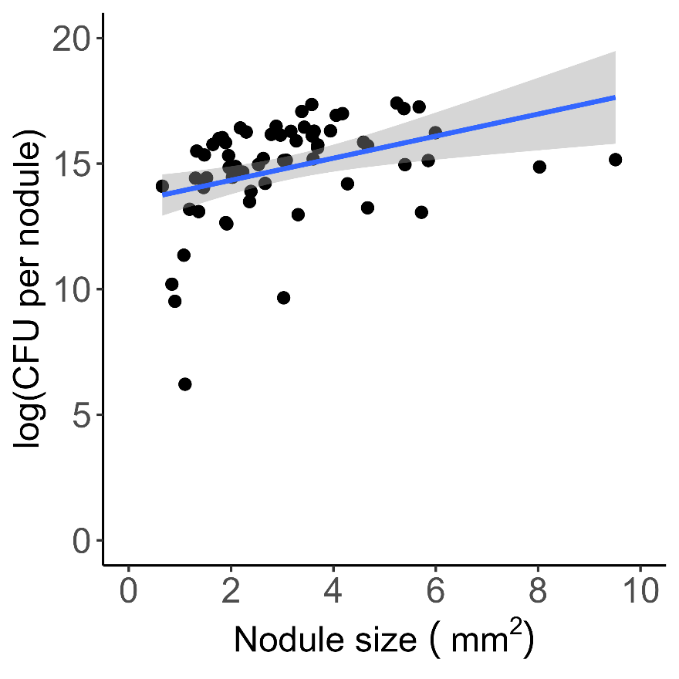


**Fig S1. Number of viable rhizobia in nodule increases with nodule size**. Nodule size data was generated by manually tracing nodule outlines from root images. Log-transformed CFU per nodule was positively correlated with nodule size (r = 0.37, n = 65 nodules, *P* = 0.00237).


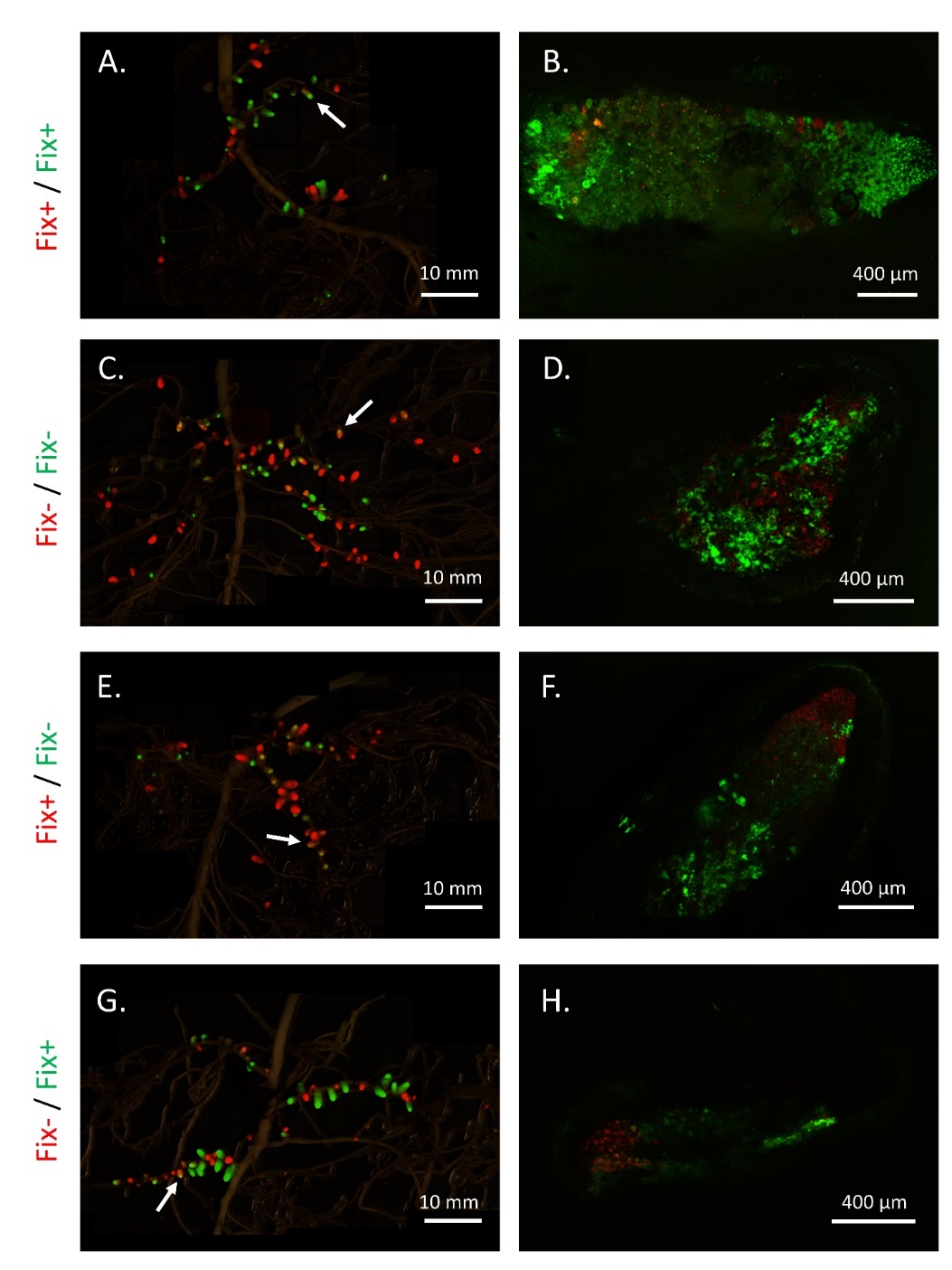


**Fig S2. Fluorescent markers enable detection of nodules founded by Fix+, Fix-, or both rhizobia strains on pea plants.** Plants were inoculated with red and green Fix+ rhizobia (**A, B**), red and green Fix- rhizobia (**C, D**), red Fix+ and green Fix- rhizobia (**E, F**), or red Fix- and green Fix+ rhizobia (**G, H**). Left-hand panels (**A, C, E, G**) show mosaic images consisting of red fluorescence, green fluorescence, and darkfield layers. White arrows indicate examples of nodules showing both red and green fluorescence (i.e., mixed-color nodules). Right-hand panels (**B, D, F, H**) show confocal images of longitudinal sections of mixed-color nodules. All panels show wild pea (see **Fig 2** for domesticated pea). For mosaic images, mean pixel brightness was approximately 2-fold greater for the red vs green layer. To improve visibility of green nodules in the mosaic images, we reduced the pixel display range to 0-50 for the green layer, leaving the red layer at the default range of 0-255. Data analysis used unaltered images.


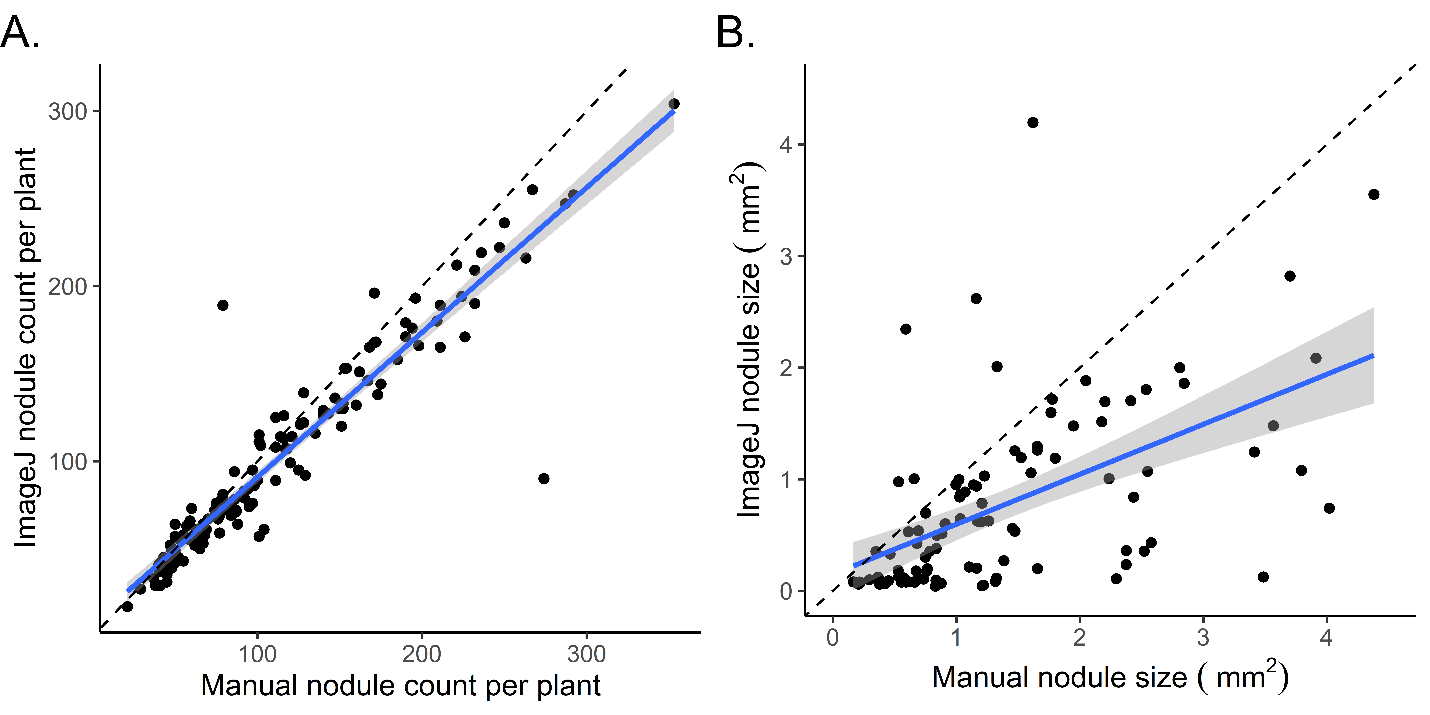


**Fig S3. Nodule traits estimated using ImageJ are positively correlated with traits measured manually from root images.** (**A**) Nodule count per plant was estimated for each nodulated plant using a custom ImageJ macro. We generated manual nodule counts per plant by counting nodules from root images. ImageJ nodule count was positively correlated with manual nodule count (r = 0.94, n = 142 plants, *P* < 0.0001). (**B**) Size of individual nodules was estimated for each of 14,259 nodules using a custom ImageJ macro. We randomly selected 100 nodules from this dataset and traced nodule outlines to generate manual nodule size data. ImageJ nodule size was positively correlated with manual nodule size (r = 0.53, n = 100 nodules, *P* < 0.0001), although the ImageJ macro underestimated nodule size, particularly for larger nodules. For both (**A**) and (**B**), the dashed diagonal line indicates a perfect fit (intercept = 0, slope = 1) between ImageJ and manual nodule trait data. Blue lines and gray shading indicate best fit slopes and confidence intervals for the relationship between ImageJ and manual nodule traits.


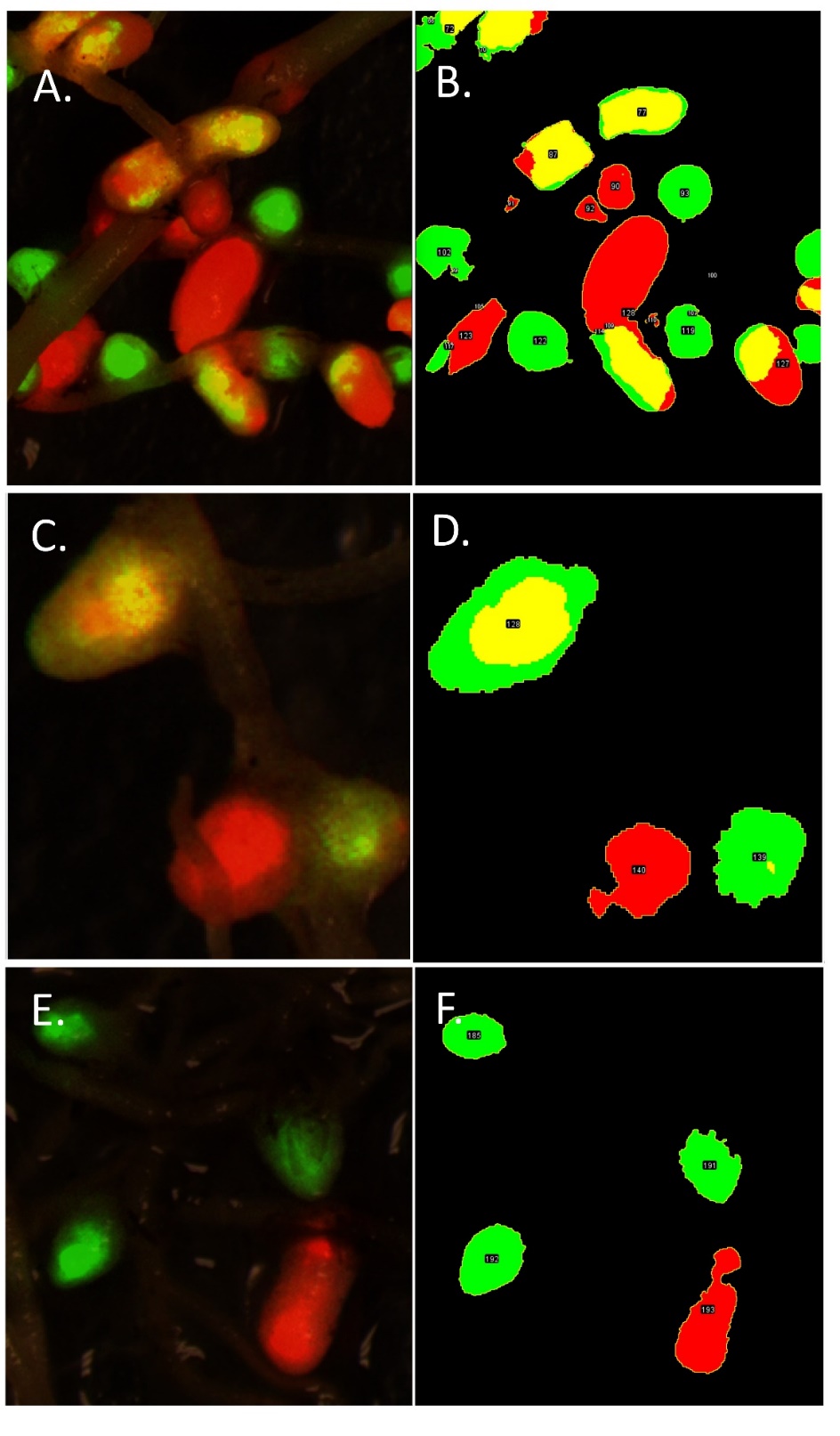


**Fig S4.** **Examples of input fluorescence images and ImageJ output showing predicted nodule color and size.** Left-hand panels show input images, and right-hand panels shows the annotated ImageJ outputs of nodule size and color (green = green fluorescence; red = red fluorescence; yellow = green and red fluorescence); numbers are unique identifiers for ImageJ features (i.e., predicted nodules or holes in nodules; holes in nodules were excluded from the final dataset). (**A-B**) Closeup of plant A158. (**C-D**) Closeup of plant A015. (**E-F**) Closeup of plant A099.


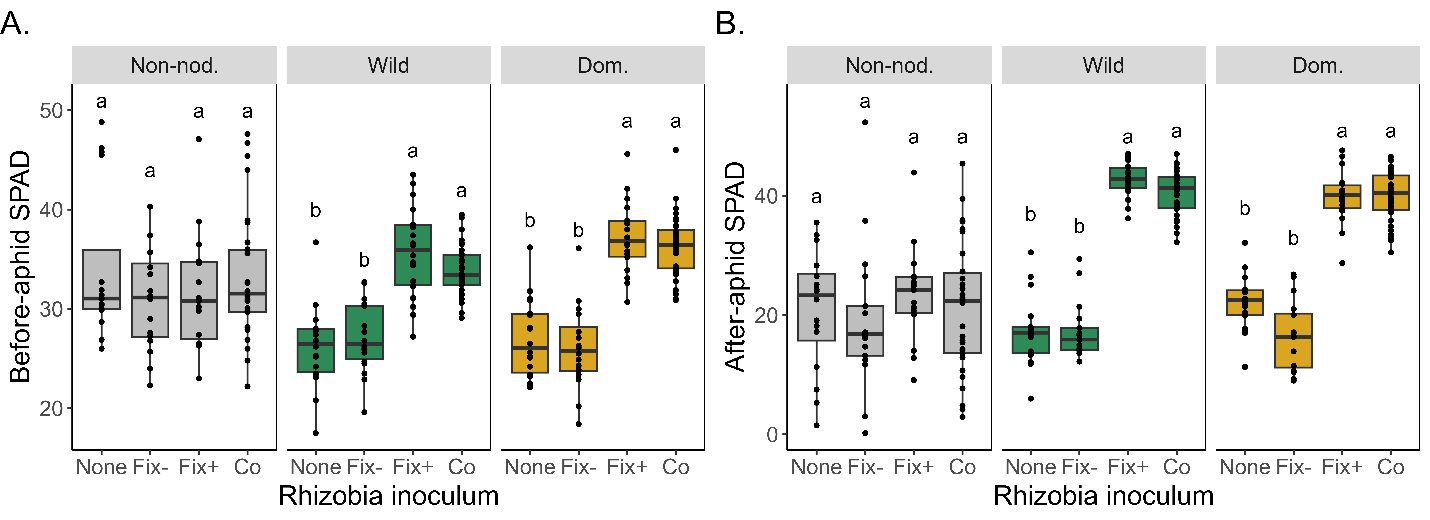


**Fig S5. Wild and domesticated pea gain similar SPAD benefits from inoculation with Fix+ rhizobia.** SPAD (Soil Plant Analysis Development) is a proxy for chlorophyll density. Wild and domesticated pea had significantly higher SPAD in the Fix+ and Co-inoculation treatments compared to the Fix- treatment both (**A**) before aphid exposure (Accession x Inoculum χ^2^(6) = 60.0, n = 255, *P* = 5.19e-11; see **Table S2**) and (**B**) after aphid exposure (Accession x Inoculum χ^2^(6) = 123, n = 255, *P* < 2e-16; see **Table S2**). For non-nodulating pea, SPAD did not vary among inoculum treatments either before or after aphid exposure. “Co” refers to co-inoculation (i.e., 2-strain inoculation.) For both **(A)** and **(B)**, each dot shows data from a single plant, the box shows the treatment median ± 1 quartile, and the whiskers encompass all datapoints within 1.5 times the interquartile range of the box. “Co” refers to the co-inoculation treatment (i.e., 2-strain inoculation), which had twice the replication of the other treatments due to pooling the reciprocally labeled co-inoculation treatments. Different letters indicate significantly different means among inoculation treatments within each pea accession. Post-hoc explorations used a significance threshold of α = 0.05 and a Holm *P-*value correction for multiple comparisons.


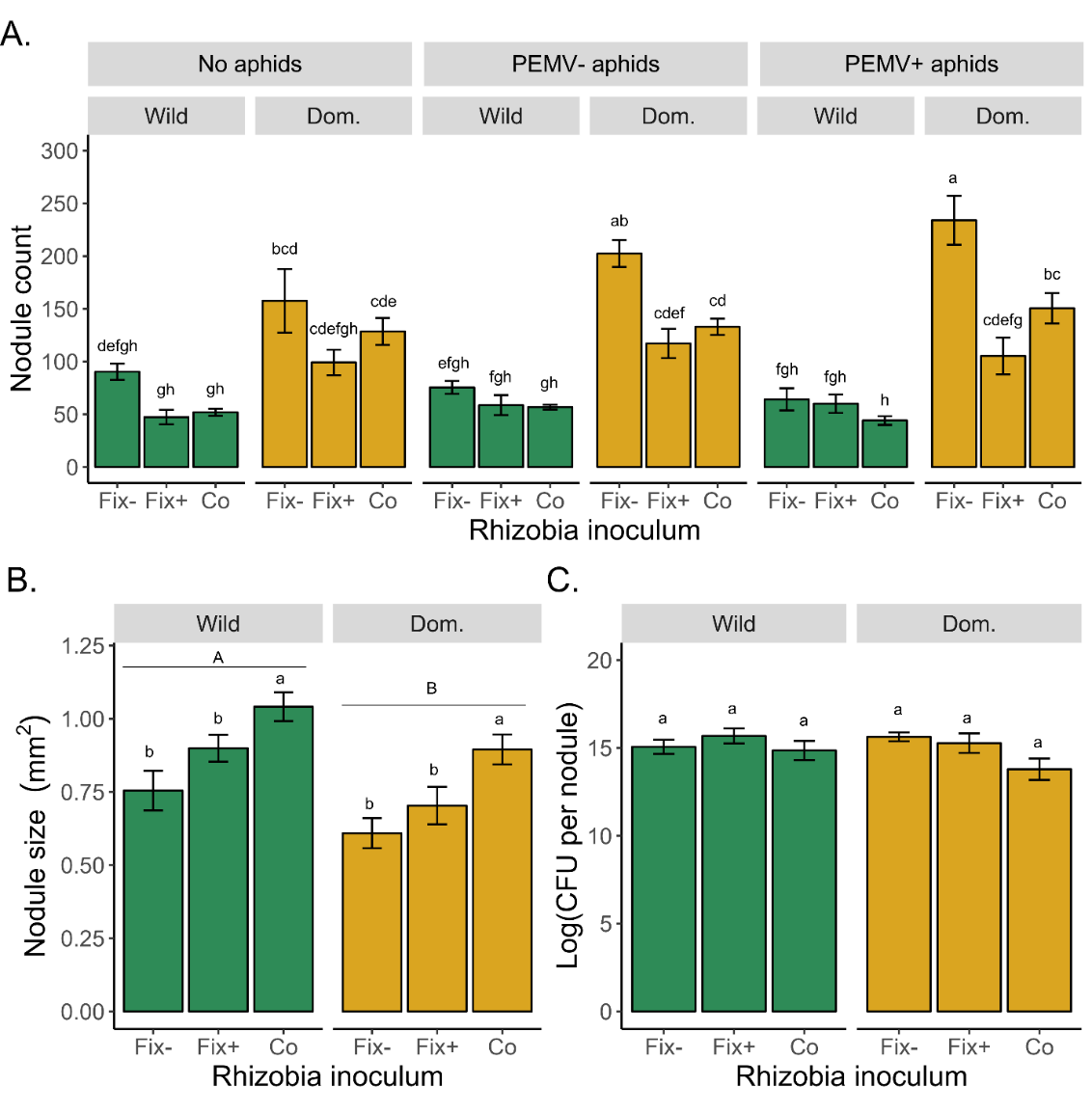


**Fig S6. Wild pea forms fewer and larger nodules than domesticated pea, but accessions have similar rhizobial CFU per nodule.** (**A**) Nodule count per plant varied significantly among inocula for each pea accession and aphid treatment combination (Accession x Inoculum x Aphid χ^2^(4) = 10.9, n = 142, *P* = 0.0272; see **Table S6**): when exposed to PEMV- or PEMV+ aphids, domesticated pea formed more nodules with the Fix- treatment than with the Fix+ or co-inoculation treatments. In contrast, domesticated pea not exposed to aphids formed similar numbers of nodules in each inoculation treatment, and wild pea formed similar numbers of nodules in all inoculation and aphid treatments. (**B**) Mean nodule size was greater for wild pea than domesticated pea (Accession χ^2^(1) = 15.1, n = 142, *P* = 1.02e-4; see **Table S6**). Nodules from co-inoculated plants were larger than nodules from plants inoculated with Fix- or Fix+ rhizobia alone (Inoculum χ^2^(2) = 32.3, n = 142, *P* = 9.46e-8; see **Table S6**). (**C**) Number of viable rhizobia in cultured nodules did not vary among inocula or between accessions (**Table S6**). “Co” refers to co-inoculation (i.e., 2-strain inoculation.) Bars indicate +/- 1 SE. Different letters indicate significantly different means. Post-hoc explorations used a significance threshold of α = 0.05 and a Holm *P-*value correction for multiple comparisons.

**Table S1. PCR primers used to amplify PEMV.**

| **Gene** | **Primer sequences (5’-3’)** | **Amplicon size (bp)** |
| --- | --- | --- |
| PEMP CP Fp  PEMP CP Rp | GTGGTGGCACCCTCTATG  GTGTCCACATGGTAGGCTATG | 290 |

**Table S2. GLMM statistics testing how plant benefits from rhizobia vary by pea accession, rhizobial inoculum, and aphid treatment (see Fig 3 and Fig S5).** “Accession” indicates the pea accession (non-nodulating, wild, or domesticated pea). “Inoculum” indicates the rhizobial treatment (None, Fix-, Fix+, or Co-inoculation, for which we pooled two reciprocally labeled 2-strain treatments). “Aphid” indicates the aphid treatment (None, PEMV- aphids, or PEMV+ aphids). Aphid terms were not tested for before-aphid SPAD, since data were collected before the start of the aphid treatment, or for PEMV titer, since all PEMV data came from plants in the same aphid treatment. We excluded two outliers (plants A063 and A192) from the PEMV titer dataset, since they had values more than 5 SD above the mean. For each term of interest, we report likelihood ratio test *χ^2^* statistics with degrees of freedom in parentheses. *** *P* < 0.0001. ** *P* < 0.001. * *P* < 0.05. ns = nonsignificant. *NA* = term not tested.

|  | Log(Shoot mass, g) | Before-aphid SPAD | After-aphid SPAD | PEMV titer |
| --- | --- | --- | --- | --- |
|  | *N = 255* | *N = 255* | *N = 255* | *N = 68* |
| Accession | χ^2^(2) = **268***** | χ^2^(2) = **6.24*** | χ^2^(2) = **77.9***** | χ^2^(2) = 0.200 (ns) |
| Inoculum | χ^2^(3) = **82.5***** | χ^2^(3) = **91.6***** | χ^2^(3) = **188***** | χ^2^(3) = **12.2*** |
| Aphid | χ^2^(2) = 2.30 (ns) | *NA* | χ^2^(2) = 0.811 (ns) | *NA* |
| Acc × Inoc | χ^2^(6) = **75.3***** | χ^2^(6) = **60.0***** | χ^2^(6) = **123***** | χ^2^(6) = 9.17 (ns) |
| Acc × Aphid | χ^2^(4) = 7.11 (ns) | *NA* | χ^2^(4) = 6.42 (ns) | *NA* |
| Inoc × Aphid | χ^2^(6) = 5.89 (ns) | *NA* | χ^2^(6) = 2.06 (ns) | *NA* |
| Acc × Inoc × Aphid | χ^2^(12) = 5.88 (ns) | *NA* | χ^2^(12) = 6.33 (ns) | *NA* |
| (1\|Block) | χ^2^(1) = **20.2***** | **χ^2^(1) = 29.1***** | χ^2^(1) = **54.4***** | χ^2^(1) < 0.0001 (ns) |

**Table S3. GLMM statistics testing how the cross-sectional area of nodules varies by pea accession, inoculum type, nodule occupant, and aphid treatment (see Fig 4a).** Only data from single-occupant nodules (n = 13219 nodules from 142 plants) were analyzed. “Accession” indicates the pea accession (wild or domesticated pea). “Inoculum” indicates the rhizobial treatment (1-strain or 2-strain). “Occupant” indicates the occupant of the nodule (Fix- or Fix+). “Aphid” indicates the aphid treatment (no aphids, PEMV- aphids, or PEMV+ aphids). For each term of interest, we report likelihood ratio test *χ^2^* statistics with degrees of freedom in parentheses. *** *P* < 0.0001. ** *P* < 0.001. * *P* < 0.05. † *P* < 0.10. ns = nonsignificant.

|  | **Single-occupant nodule area (mm^2^)** |
| --- | --- |
|  | *N = 13219 nodules from 142 plants* |
| Accession | χ^2^(1) = **16.4***** |
| Inoculum | χ^2^(1) = **4.73*** |
| Occupant | χ^2^(1) = **610***** |
| Aphid | χ^2^(2) = 1.01 (ns) |
| Accn:Inoc | χ^2^(1) = 0.319 (ns) |
| Accn:Occupant | χ^2^(1) = 2.06 (ns) |
| Accn:Aphid | χ^2^(2) = 1.57 (ns) |
| Inoc:Occupant | χ^2^(1) = **59.8***** |
| Inoc:Aphid | χ^2^(2) = 2.77 (ns) |
| Occupant:Aphid | χ^2^(2) = 0.861 (ns) |
| Accn:Inoc:Occupant | χ^2^(1) = 0.124 (ns) |
| Accn:Inoc:Aphid | χ^2^(2) = 0.679 (ns) |
| Accn:Occupant:Aphid | χ^2^(2) = **8.56*** |
| Inoc:Occupant:Aphid | χ^2^(2) = 0.847 (ns) |
| Accn:Inoc:Occupant:Aphid | χ^2^(2) = 0.0522 (ns) |
| (1\|Block) | χ^2^(1) = **26.4***** |
| (1\|Plant) | χ^2^(1) = **353***** |

**Table S4. GLMM statistics testing how the cross-sectional area occupied by strains in single-infection nodules and mixed-infection nodules varies by nodule occupant, pea accession, and aphid treatment during 2-strain inoculations (see Fig 4b-c).** The cross-sectional area occupied by a strain in a nodule was used as the basis of measuring host sanctions. To test for among-nodule sanctions, we analyzed total nodule area of 5740 single-infection nodules (i.e., containing just the Fix+ or Fix- strain) from 72 plants during 2-strain inoculations. To test for within-nodule sanctions, we analyzed partial nodule area of 1040 mixed-infection nodules from 72 plants during 2-strain inoculations; each nodule provided two observations (partial nodule size for Fix+ and Fix- strain). “Occupant” indicated the strain in the nodule (Fix+ or Fix-) and the significance of this term (and any interactions including “Occupant”) provided the statistical test for sanctions occurring. “Accession” indicated the pea genotype (wild or domesticated). “Aphid” indicated the aphid treatment (no aphids, PEMV- aphids, or PEMV+ aphids). “Marker” indicated the fluorescent label of the Fix+ strain on the plant (green or red). “Plant” and “Nodule” identified individual plants or nodules, respectively. For each term of interest, we report likelihood ratio test *χ^2^* statistics with degrees of freedom in parentheses. *** *P* < 0.0001. ** *P* < 0.001. * *P* < 0.05. † *P* < 0.10. ns = nonsignificant. *NA* = term not tested.

|  | **Among-nodule sanctions:**  **Log(Total nodule area, mm^2^)** | **Within-nodule sanctions:**  **Log(Partial nodule area, mm^2^)** |
| --- | --- | --- |
|  | *N = 5740 nodules from 72 plants* | *N = 1040 nodules from 72 plants* |
| Occupant | χ^2^(1) = **294***** | χ^2^(1) = 0.00816 (ns) |
| Accession | χ^2^(1) = **6.15*** | χ^2^(1) = 1.26 (ns) |
| Aphid | χ^2^(2) = 0.310 (ns) | χ^2^(2) = 2.19 (ns) |
| Occupant × Accn | χ^2^(1) = 0.0505 (ns) | χ^2^(1) = 0.0422 (ns) |
| Occupant × Aphid | χ^2^(2) = 0.846 (ns) | χ^2^(2) = **14.7**** |
| Accn × Aphid | χ^2^(2) = 2.78 (ns) | χ^2^(2) = 0.180 (ns) |
| Occupant × Accn × Aphid | χ^2^(2) = **18.0**** | χ^2^(2) = 1.71 (ns) |
| Marker | χ^2^(1) = 3.34† | χ^2^(1) = 0.825 (ns) |
| (1\|Block) | χ^2^(1) = **10.0*** | χ^2^(1) = **11.3**** |
| (1\|Plant) | χ^2^(1) = **64.1***** | χ^2^(1) = **11.5**** |
| (1\|Nodule) | *NA* | χ^2^(1) = ~0 (ns) |

**Table S5. GLMM statistics testing how plant PEMV titer varies based on the strictness of host sanctions and pea accession (see Fig 5).** Strictness of among-nodule sanctions and within-nodule sanctions were measured as log(Fix+ nodule area/Fix- nodule area) using single-infection or mixed-infection nodules, respectively. All analyses used data from 20 plants exposed to PEMV+ aphids during 2-strain inoculations with Fix+ and Fix- rhizobia. For each term of interest, we report likelihood ratio test *χ^2^* statistics with degrees of freedom in parentheses. *** *P* < 0.0001. ** *P* < 0.001. * *P* < 0.05. † *P* < 0.10. ns = nonsignificant.

|  |  | **PEMV titer** |
| --- | --- | --- |
| Among-nodule sanctions model | | |
|  | Strictness of sanctions | χ^2^(1) = 0.649 (ns) |
|  | Accession | χ^2^(1) = 2.26 (ns) |
|  | Strictness × Accession | χ^2^(1) = 0.188 (ns) |
|  | Marker | χ^2^(1) = 0.079 (ns) |
|  | (1\|Block) | χ^2^(1) = 0.163 (ns) |
| Within-nodule sanctions model | | |
|  | Strictness of sanctions | χ^2^(1) = **3.98*** |
|  | Accession | χ^2^(1) = 2.06 (ns) |
|  | Strictness × Accession | χ^2^(1) = 0.00570 (ns) |
|  | Marker | χ^2^(1) = 0.936 (ns) |
|  | (1\|Block) | χ^2^(1) = 0.00622 (ns) |

**Table S6. GLMM statistics testing how nodule count, nodule cross-sectional area, and number of culturable rhizobia per nodule vary by accession, inoculum, and aphid treatments (see Fig S6).** “Accession” indicates the pea genotype (wild or domesticated pea). “Inoculum” indicates the rhizobial treatment (Fix+, Fix-, or co-inoculated, for which we pooled the two reciprocally labeled 2-strain treatments). “Aphid” indicates the aphid treatment (no aphids, PEMV- aphids, or PEMV+ aphids). Likelihood ratio test *χ^2^* statistics are reported. *** *P* < 0.0001. ** *P* < 0.001. * *P* < 0.05. † *P* < 0.10. ns = nonsignificant. *NA* = term not tested.

|  | Nodule count | Total nodule area (mm^2^) | Log(CFU per nodule) |
| --- | --- | --- | --- |
|  | *N = 142* | *N = 142* | *N = 65* |
| Accession | χ^2^(1) = **134***** | χ^2^(1) = **15.1**** | χ^2^(1) = 1.21 (ns) |
| Inoculum | χ^2^(2) = **45.2***** | χ^2^(2) = **32.3***** | χ^2^(2) = 5.37† |
| Aphid | χ^2^(2) = 3.05 (ns) | χ^2^(2) = 1.66 (ns) | χ^2^(2) = 1.22 (ns) |
| Acc × Inoc | χ^2^(2) = **20.4***** | χ^2^(2) = 0.568 (ns) | χ^2^(2) = 2.49 (ns) |
| Acc × Aphid | χ^2^(2) = **8.36*** | χ^2^(2) = 1.98 (ns) | χ^2^(2) = 0.0929 (ns) |
| Inoc × Aphid | χ^2^(4) = 1.36 (ns) | χ^2^(4) = 2.85 (ns) | χ^2^(4) = 1.73 (ns) |
| Acc × Inoc × Aphid | χ^2^(4) = **10.9*** | χ^2^(4) = 2.20 (ns) | χ^2^(4) = 0.760 (ns) |
| (1\|Block) | χ^2^(1) = 0.285 (ns) | χ^2^(1) = 32.4*** | *NA* |
| (1\|Plant) | *NA* | *NA* | χ^2^(1) = ~0 (ns) |

**Methods S1.** **Culturing rhizobia from nodules**

**Isolating nodules.** We cultured nodules from 24 plants in block 4 (2 nodulating plant genotypes (i.e., wild or domesticated pea) × 3 aphid treatments × 4 inoculation treatments = 24 plants). From each plant, we selected up to 4 nodules (1 pure red, 1 pure green, 1 mixed red and green, and 1 non-glowing), for a maximum of 24 × 4 = 96 nodules. Nodules for culturing were selected based on how easy it was to separate the nodule from the plant without damaging the nodule. Nodules were individually photographed before removal from the root, and we noted the level of magnification for each image to allow calculations of nodule size based on the known scale of the main root images (which had a 1cm grid in the background). We harvested nodules from 8 plants per day from 1-3 March 2021, working on plants from a single aphid treatment each day. Nodules were stored in sterile water in a flat-bottom 96-well plate at 4°C.

**Surface-sterilizing nodules**. On 4 March 2021, nodules were transferred to wells of the “nodulizer” (a flat-bottom 96-well plate with lid and bottom replaced by fine mesh to allow liquids to pass through wells). The nodulizer was manually agitated in germicidal bleach for 90 seconds, followed by 3 × 60 second rinses in sterile water. Due to problems with the nodulizer lid, a few nodules fell out of their wells and were lost, while other nodules were accidentally bumped into other wells. Two wells that contained two nodules were renamed with an “X” prefix; we attempted to identify these later based on photos taken of the nodules after surface sterilization and photos of the nodules on the roots.

**Crushing nodules and checking for rhizobial viability**. Surface-sterilized nodules were immediately transferred to individual labeled epitubes containing 100µL sterile MAG + Neo. Nodules were crushed with sterile pestles. To check that nodules contained viable bacteria, we plated 20uL of undiluted nodule slush onto MAG + Neo agar plates and checked plates for growth on 12 days after plating (16 March 2021). Results are shown below. Most of the red, green, and mixed nodules produced growth, but none of the non-glowing nodules grew, which suggests that non-glowing nodules were senescing and lacked viable rhizobia. If the non-glowing nodules had grown on plates, this would have suggested that plants were forming nodules with strains that lacked fluorescent markers.

| Anticipated nodule color | # nodules that did grow | # nodules that did not grow | # nodules contaminated; could not score | Total # nodules |
| --- | --- | --- | --- | --- |
| Green only | 16 | 3 | 1 | 20 |
| Red only | 17 | 5 | 1 | 23 |
| Mixed red and green | 26 | 3 | 1 | 30 |
| Nonglowing | 0 | 14 | 0 | 14 |
| Total | 59 | 25 | 3 | 87 |

**Diluting and drop-plating nodules.** Immediately after crushing nodules, we transferred 40µL crushed nodule to 160µL MAG + Neo (representing 40% of the total CFUs in the nodule, with dilution factor = 2.5). We then performed 7 additional serial dilutions by transferring 40µL CFU-containing dilution into 160µL MAG + Neo (representing 20% of the previous step, with dilution factor = 5). For the final 6 dilutions, which we referred to by the letters C-H, we plated 25µL drops (representing 12.5% of the previous step, with dilution factor = 8) onto MAG + Neo agar. Thus, the final dilution factors for the plated dilutions were as follows:

| Dilution | Calculation of dilution factor | Overall dilution factor |
| --- | --- | --- |
| C | 2.5*(5*5)*8 | 500 |
| D | 2.5*(5*5*5)*8 | 2500 |
| E | 2.5*(5*5*5*5)*8 | 12500 |
| F | 2.5*(5*5*5*5*5)*8 | 62500 |
| G | 2.5*(5*5*5*5*5*5)*8 | 312500 |
| H | 2.5*(5*5*5*5*5*5*5)*8 | 1562500 |

We plated 3 technical replicates of each dilution, so that each nodule had up to 18 drops (6 dilutions × 3 technical replicates). All nodule dilutions were plated on the same day (4 March 2021). We used a Sharpie to mark the positions of individual colonies within each drop during the week of 8 March 2021; these dots were later counted and recorded. On 19 March 2021, we checked each drop under the Leica M165 FC Fluorescent Stereo Microscope and recorded whether there were only green colonies, only red colonies, a mixture of green and red, or no growth.

**Estimating CFU per nodule**. From colony counts for each plated drop, we estimated the number of CFU in the original nodule by multiplying colony counts by the relevant dilution factor. We ignored colony counts greater than 50, since these were unlikely to be reliable given the small area covered by the drop.

**Methods S2. Developing and checking ImageJ macro for estimating nodule traits**

**Assembling stitched images of intact root systems.** Using a Leica M165 FC Fluorescent Stereo Microscope, we generated three images for each field of view: darkfield, green fluorescence, and red fluorescence. Each plant’s root system was covered by multiple fields of view. Raw images were imported into Microsoft PowerPoint and manually resized, positioned, and overlapped to form a mosaic image for each plant. Each plant produced three mosaic images (darkfield, green, and red), which were exported as high-resolution TIFFs. Because the original images were resized in PowerPoint, which was done separately for each plant, the mosaic images for each plant differ in their resolution from the original raw images. However, each mosaic darkfield image had a 1cm grid in the background of the image, and we used ImageJ to record the number of pixels in a 2cm section of the grid. From this data, we calculated pixels per mm^2^ for each plant’s set of mosaic images, enabling us to convert pixel counts (gathered below) to dimensions in mm^2^.

**Custom ImageJ macro to gather data on individual nodules.** We prepared a custom ImageJ macro to evaluate the mosaic images for each plant (“NodCounts_v3.ijm”). The purpose of this macro was to estimate nodule size and relative amounts of red and green fluorescence for each nodule on each plant. We noticed that red mosaic images were about 2-fold brighter than green images. To attempt to equalize our ability to detect both red and green nodules, the macro used a higher threshold for detecting red fluorescence and a lower threshold for detecting green fluorescence. The macro generated the following data for each predicted nodule on each plant: its size in pixels, percentage of the nodule that was green, percentage of the nodule that was red, and a unique identifier that allowed us to check on particular nodules visually. The nodule size data had a strong right skew due to the macro sometimes clustering multiple nodules into single predicted features. Furthermore, at the small end of the nodule size range, predicted features were often fragments of nodules (i.e., where multiple features matched to the same nodule).

**Data curation in R: Dropping erroneous features.** The ImageJ macro identified 23,283 features on the 142 nodulated plants in the experiment. Some of the features were not nodules, but instead were holes in nodules—identifiable by having a positive size in pixels but being zero percent red and zero percent green. We dropped all features that were holes in nodules. Some features had holes in them, and these holes were filled with the surrounding color. Finally, some features were just flecks within holes within nodules, and these flecks were deleted. After making these corrections, the dataset contained 21,182 features, all classified as “pure green”, “pure red”, or “mixed.”

**Data curation in R: Setting thresholds for maximum and minimum nodule size.** We next attempted to exclude erroneous features that were either clusters of nodules or fragments of nodules, rather than single entire nodules. Thus, we set minimum and maximum nodule size thresholds on the nodule data to exclude features that were unlikely to represent single nodules. We aimed to include as many predicted features as were biologically feasible in the final dataset. To identify the threshold for maximum nodule size, we sorted the predicted nodules by size (mm^2^) and visually inspected them in descending order. The largest features were clusters of nodules and we proceeded with visual inspection until finding a feature that represented only a single nodule. This was nodule 51 of plant A254, which had a size of 7.61 mm^2^. We excluded the 59 features larger than this cutoff, leaving 21,123 features in the dataset. There were many features in the dataset with extremely small sizes (i.e., 113 features composed of 1 pixel; 1102 features composed of 2 pixels), so it was not feasible to visually inspect all nodules in size order as we had done for the large nodules. Instead, we inspected nodules on the plant whose mosaic images had the highest resolution (i.e., smallest pixels) and found the smallest feature on the plant that identified an entire nodule, rather than a fragment of a nodule. This was nodule 142 of plant A033, with a pixel count of 248 and a size of 0.04 mm^2^. Because this plant had higher resolution than any other plant, 248 pixels for plant A033 was a smaller nodule than 248 pixels for any other plant (the size of a 248-pixel nodule ranged from 0.04 mm^2^ to 0.14 mm^2^ across all plants in the dataset). Thus, a minimum size threshold of 0.04 mm^2^ seemed likely to keep all real nodules in the dataset. We excluded 6,864 features smaller than this cutoff, leaving 14,259 features in the dataset. Overall, our approach made it unlikely that we excluded any real single nodules, although we erred on the side of also including some erroneous features. We expect this decision would have introduced some unbiased noise to the data, making it more difficult to detect small but real effects of our experimental manipulations.

**Checking accuracy of nodule count estimates.** To check the accuracy of nodule counts generated by the ImageJ macro, we overlaid the mosaic images for each plant (red, green, darkfield) using another custom macro (“Overlays.ijm”). We used the ImageJ plugin “Cell Counter” to annotate these images with markers corresponding to red, green, mixed, and nonglowing nodules. We extracted the total number of manually counted nodules for each plant and compared them to estimates from the NodCounts_v3 macro (see [**Fig S3a**](#FigS2)).

**Checking the accuracy of nodule size estimates.** We made another custom macro (“Nodcounts v3 darkfield sizes.ijm”) to overlay darkfield root images with annotated output images from the NodCounts_v3 macro. Then, we randomly selected 100 nodules from the dataset of 14,259 nodules and manually traced nodule circumferences in ImageJ. Of the 100 randomly selected nodule ID’s, 78 were single nodules. Fifteen nodule ID’s corresponded to nodule fragments (i.e., formed a single nodule only if added to another nodule ID); for these, we obtained manual size data by tracing the entire nodule visible in the darkfield image, rather than just the fragment identified by that nodule ID. Seven nodule ID’s corresponded to multiple nodules (i.e., formed a single nodule only if split up into 2 or more pieces); for these, we obtained manual size data by tracing a representative nodule within the nodule cluster. We extracted the manually estimated nodule size data and compared them to estimates of nodule size from the NodCounts_v3 macro (see [**Fig S3b**](#FigS2)). ImageJ calculated nodule sizes were a median of 55% smaller than manual traces ([**Fig S**](#FigS5)**4**).
